# Distinctive acidity in citrus fruit is linked to loss of proanthocyanidin biosynthesis

**DOI:** 10.1016/j.isci.2024.110923

**Published:** 2024-09-13

**Authors:** Elliott Atkins, Emanuele Scialò, Chiara Catalano, Carmen Caballero Hernández, Eva Wegel, Lionel Hill, Concetta Licciardello, Leandro Peña, Andrés Garcia-Lor, Cathie Martin, Eugenio Butelli

**Affiliations:** 1 John Innes Centre, Norwich NR4 7UH, UK; 2 CREA, Research Center for Olive Fruit and Citrus Crops, Corso Savoia 190, 95024 Acireale, Italy; 3 Instituto de Biologia Molecular y Celular de Plantas – Consejo Superior de Investigaciones Científicas, Universidad Politécnica de Valencia, Valencia, Spain; 4 Centro de Citricultura y Producción Vegetal, Instituto Valenciano de Investigaciones Agrarias, Valencia, Spain

**Keywords:** molecular biology, plant biology, plant physiology

## Abstract

The distinctive acidity of citrus fruit is determined by a regulatory complex of MYB and bHLH transcription factors together with a WDR protein (MBW complex) which operates in the unique juice vesicles of the fruit. We describe a mutation affecting the MYB protein, named Nicole, in sweet orange and identify its target genes that determine hyperacidification, specifically. We propose that the acidity, typical of citrus fruits, was the result of a loss of the ability of Nicole to activate the gene encoding anthocyanidin reductase, an enzyme essential for the synthesis of proanthocyanidins, which are absent in citrus fruit.

## Highlights

• *Nicole i*s essential for the regulation of fruit acidity in citrus
• In a natural low-acid mutant of sweet orange, *Nicole* is interrupted by a retrotransposon
• Nicole upregulates vacuolar proton pumps and several proanthocyanidin biosynthetic genes
• Incomplete activation of the proanthocyanidin pathway may explain extreme acidity in citrus

## Graphical abstract

**Figure:**
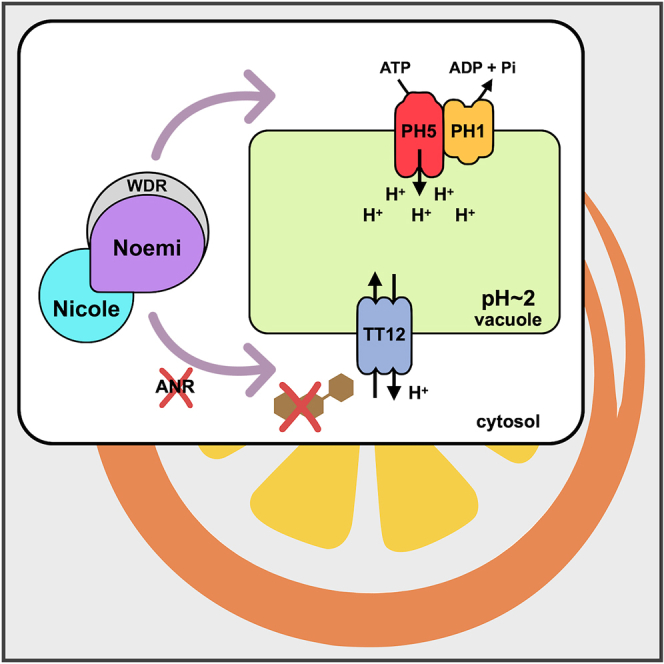

## Teaser

Molecular biology; Plant biology, Plant Biology, Plant physiology

## Introduction

Citrus fruits are well known for their acidity and, with a pH of around 2, lemon is the most acidic common constituent of the human diet. Many wild and cultivated citrus species and hybrids (including citron, most limes, “true” mandarins, trifoliate, and sour orange) share similarly high levels of acidity. The acidity of citrus fruit may have underpinned early uses as medicine and antiseptics. However, thousands of years of domestication have resulted in accessions with moderate acidity with a typical pH of around 3.5 for some mandarins and sweet orange fruit.

Together with total soluble sugars, acidity is the major determinant of taste and quality of citrus fruit. The very low pH reflects the content of citric acid (citrate), which is produced within the juice vesicles, the unique structures that form the edible part of citrus fruit.^1^^,^^2^ At the cellular level, citrate biosynthesis requires the coordinated activity of many enzymes in different compartments. It is generally assumed that reduced acidity was a key trait for citrus domestication, and genetic differences in citrate biosynthetic genes may explain differences in acidity between wild and cultivated citrus and between lemon and orange.^3^^,^^4^

**Figure 1:**
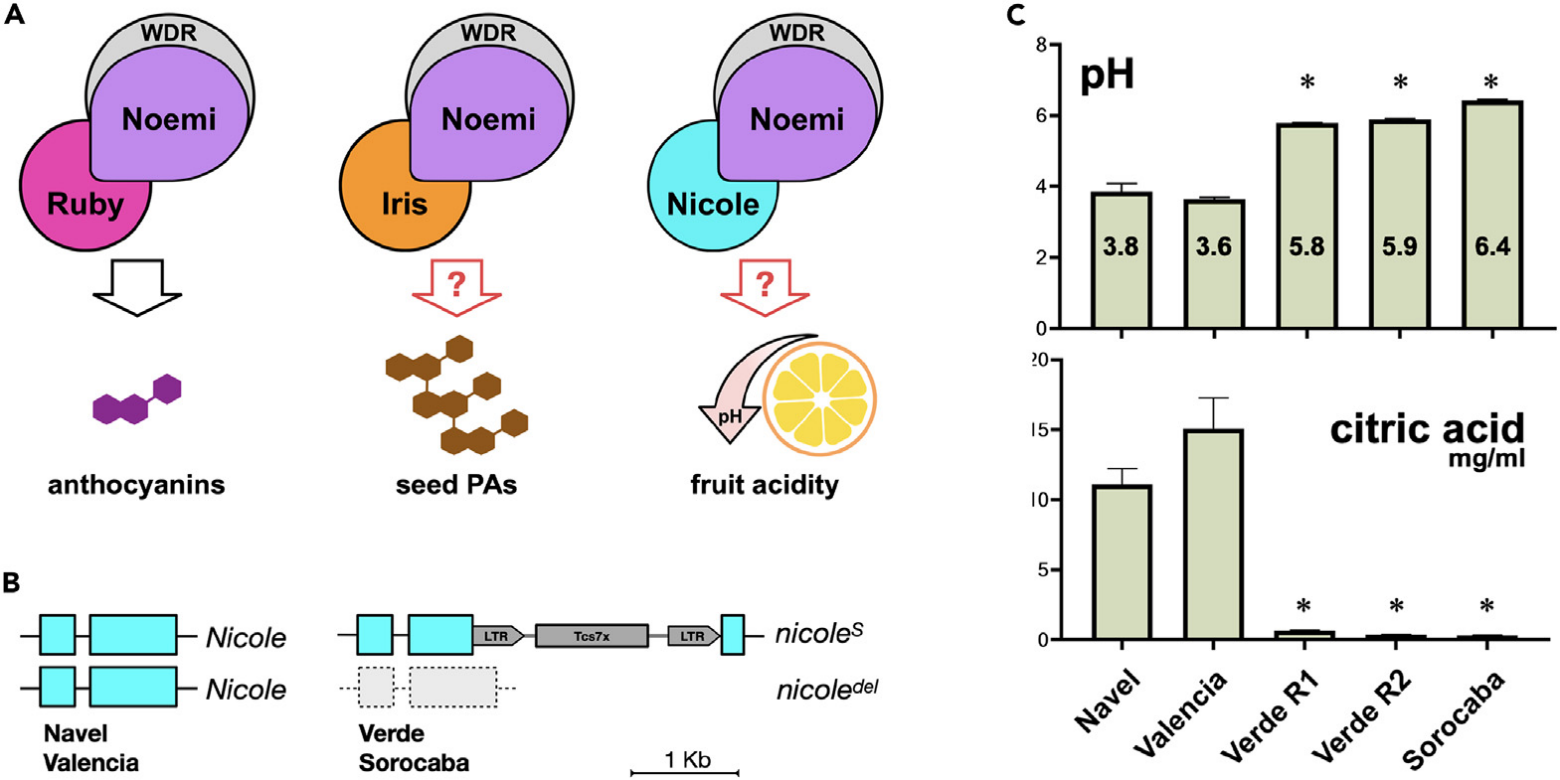
Mutations in *Nicole* are associated with very low fruit acidity and dramatic reduction in citric acid content of sweet oranges (A) Working hypothesis showing how the same bHLH (Noemi) and WDR proteins can form three distinct MBW complexes with different MYB transcription factors (Ruby, Iris, and Nicole), driving the expression of different sets of genes and regulating different physiological processes. The red question marks indicate the previously unconfirmed identities of the MYB transcription factors controlling PA accumulation in seeds or fruit acidity. (B) Allelic constitution of *Nicole* in sweet orange varieties “Navel” and “Valencia” (both wild type for acidity) or “Lima Verde” and “Lima Sorocaba”. (C) Measurement of pH and citric acid content in juice obtained from individual fruits of different varieties of sweet orange. Error bars represent the standard deviation of the mean (*n* ≥ 3). Asterisks indicate comparative statistical significance relative to “Navel”: ∗ *p* < 0.0001. See also Figures S1 and S2 and Data S1.

Besides biosynthesis and degradation, a key step in citrate metabolism is its transport to the vacuole. The current understanding is that this process is achieved through a mechanism referred to as the “acid trap,” dependent on the pH of the vacuole and the electrochemical H^+^ gradient across the tonoplast.^5^^,^^6^ Acidification of the vacuole can be achieved through the activity of vacuolar pyrophosphatases and vacuolar ATPases, proton pumps ubiquitous in plants. In some species including citrus, however, a second distinct mechanism has been identified which requires the activity of specific P-type ATPases involved in proton transport. These P-type ATPases are essential for hyperacidification, driving the pH to values considerably lower than in a typical plant vacuole (pH of around 5.5). In petunia, two P-type ATPases (named PH5 and PH1) interact functionally to induce vacuolar hyperacidification and mutants were identified by variation in pH-dependent flower color.^7^ The process of hyperacidification is controlled by a transcriptional activation complex formed by an MYB (M) transcription factor, a bHLH (B) transcription factor, and a WD-repeat (W) protein, also involved in regulating proanthocyanin and anthocyanin biosynthesis.^8^^,^^9^ Despite their broad evolutionary distance, a similar mechanism appears to control pH in citrus fruit, where the expression of *PH5* correlates with the level of acidity.^10^^,^^11^ Evidence for a conserved regulatory mechanism emerged with the genetic characterization of the “acidless” phenotype,^12^^,^^13^ observed in a peculiar group of citrus mutants where the complete lack of anthocyanins in young leaves and flowers is accompanied by the absence of proanthocyanidins (PAs; also known as condensed tannins) in seeds and, most notably, with an extreme reduction in fruit acidity (involving about two- or three-unit changes in pH compared to the corresponding wild-type accessions). These distinct physiological processes are controlled by a gene with pleiotropic functions encoding a bHLH transcription factor (named Noemi or CitAN1), leading to the hypothesis that the same bHLH and WD-repeat proteins can form three distinct MBW complexes with distinct MYB transcription factors which provide the DNA-binding specificity that drives the MBW complex to the activation of specific promoters and, therefore, to the control of specific processes (Figure 1A).^14^ Hence, while mutations in the bHLH *Noemi* gene affect all the three processes,^12^^,^^13^ mutations in a certain MYB genes would only have consequences on one of the three specific phenotypic traits. While the MYB transcription factor, Ruby, has been unequivocally established as the MYB transcription factor that activates anthocyanin biosynthesis in citrus,^15^^,^^16^^,^^17^ the identity of the genes encoding the other two MYB proteins remains uncertain. The aim of this study was the identification and characterization of those MYB transcription factors controlling PA production in seeds and fruit hyperacidification (Figure 1A).

## Results

### Isolation of Iris, MYB regulator of PA biosynthesis in seeds of sweet orange

A gene encoding a MYB transcription factor regulating PA biosynthesis in seeds, *TT2*, has been extensively characterized in many species including Arabidopsis.^18^^,^^19^^,^^20^ In citrus, however, the ortholog of *TT2* contains mutations including a G/A transition in the start codon conserved in all primary species (mandarin, pummelo, and citron) according to the citrus pangenome (http://citrus.hzau.edu.cn/). We confirmed the presence of the start codon mutations and stop mutations within the first half of the coding sequence of *TT2* in citrus primary species and in several hybrids, including sweet orange. Then, we analyzed three genes encoding MYB transcription factors which, based on sequence homology and phylogenetic analysis (Figure S1A), appeared related to TT2, clustered within the same subgroup 5a, and therefore had the potential to activate PA biosynthesis. We named them Aris, Marys, and Iris (Table S1). Marys was the most closely related to TT2 and annotated as such in the sweet orange v1.1 Phytozome reference genome (https://phytozome-next.jgi.doe.gov/info/Csinensis_v1_1). Iris was highly similar (64% amino acid identity) to VvMYBPA1, a well-characterized transcription factor that regulates PA accumulation in seeds and developing berries of grape.^21^ *Marys* and *Iris* were highly expressed in the inner seed coat isolated from different varieties of sweet orange (Figure S1B). The transcript of *Aris* was barely detectable, but we were able to isolate the corresponding full-length coding sequence from very young developing fruit. To test the functionality of the three genes, we transiently expressed their coding sequences in leaves of *N. benthamiana* and treated the extracts with DMACA (4-dimethylaminocinnamaldehyde) reagent, which specifically stains PAs and their monomers (catechins and epicatechins) a purple-blue color. Only *Iris* was able to induce the accumulation of these flavonoid compounds (Figure S1C). To confirm the functionality, or lack thereof, we used a dual luciferase reporter assay to assess the ability of Aris, Marys, and Iris to activate the promoters of anthocyanidin synthase (*ANS*), a gene essential for PA biosynthesis. Iris strongly activated this promoter, while Aris, Marys showed only very weak, most likely non-specific, activity (Figure S1D). When stably expressed in tobacco under the control of the *CaMV 35S* promoter, Iris was able to induce PA biosynthesis, particularly in flowers (Figure S1E). We concluded that Iris is the MYB controlling seed PAs in citrus (Figure 1A).

### Isolation of Nicole, MYB regulator of fruit acidity in sweet orange

By analogy to petunia, a gene encoding a MYB protein with homology to PhPH4 has been considered the regulator of fruit acidity^13^^,^^22^^,^^23^ and, recently, the use of CRISPR-Cas9 technology has resulted in edited lines of *Fortunella hindsii* (Hongkong kumquat), with substantial reduction in fruit acidity and citrate levels.^24^ To confirm the identity of the gene encoding the MYB transcription factor controlling hyperacidification of citrus fruits, we searched natural diversity for varieties with a strong reduction in fruit acidity but, unlike “acidless” pleiotropic *noemi* mutants, still able to produce PAs in seeds. Brazil is one of the world’s largest citrus producers and several varieties of sweet orange with very low acidity are relatively popular and collectively referred to as “Lima” (Figure S1F). The most common, simply named “Lima”, is a typical “acidless” variety reported as a *noemi* mutant^13^ (Figure S1H). We also examined two less common varieties, “Lima Verde” and “Lima Sorocaba” with very low fruit acidity (pH around 6) and citrate content comparable to the “acidless” *noemi* mutant (Figure 1C), but without any impairment in PA production in seeds (Figure S1G).

Sweet orange (*C. sinensis*) is an apomictic hybrid, whose complex mandarin x pummelo genetic constitution is fixed within the population. The absence of sexual recombination and its very narrow genetic base mean that the remarkable phenotypic diversity among accessions results from somatic mutations and that even single polymorphisms in a candidate gene are significant. When analyzed for genes encoding MYB proteins homologous to PhPH4, both “Lima Verde” and “Lima Sorocaba” showed the presence of a defective allele, whose coding sequence was interrupted by a partial LTR retrotransposon of 2,434 bp (Figures 1B and S2A and Data S1). We named this gene *Nicole*. No wild-type allele was detected in the mutants. The absence of a T/G polymorphism downstream of the stop codon (Figure S2B) and the localization of *Nicole* at the 5′ end of chromosome 2 (Figure S2C), in an unphased region of apparent mandarin homozygosity,^25^ suggest the presence of a terminal deletion affecting the other allele in the mutants. The retrotransposon insertion introduces a premature stop codon that prevents the transcription of the last 188 bp of the *Nicole* coding sequence, without affecting the 5′ region encoding the DNA-binding domain of the MYB factor but causing a loss of 62 amino acids which include the putative transcriptional activation domain.

### Nicole directly activates the expression of genes required for vacuolar hyperacidification and biosynthesis of proanthocyanidins, which are absent in citrus fruit

To characterize further the *nicole* mutants, we analyzed the accumulation pattern of flavonoids and phenolic acids in fruit juice. Other than a strong reduction in ferulic acid, we could not detect major differences compared to the acidic “Navel” variety (Figure S2D).

**Figure 2:**
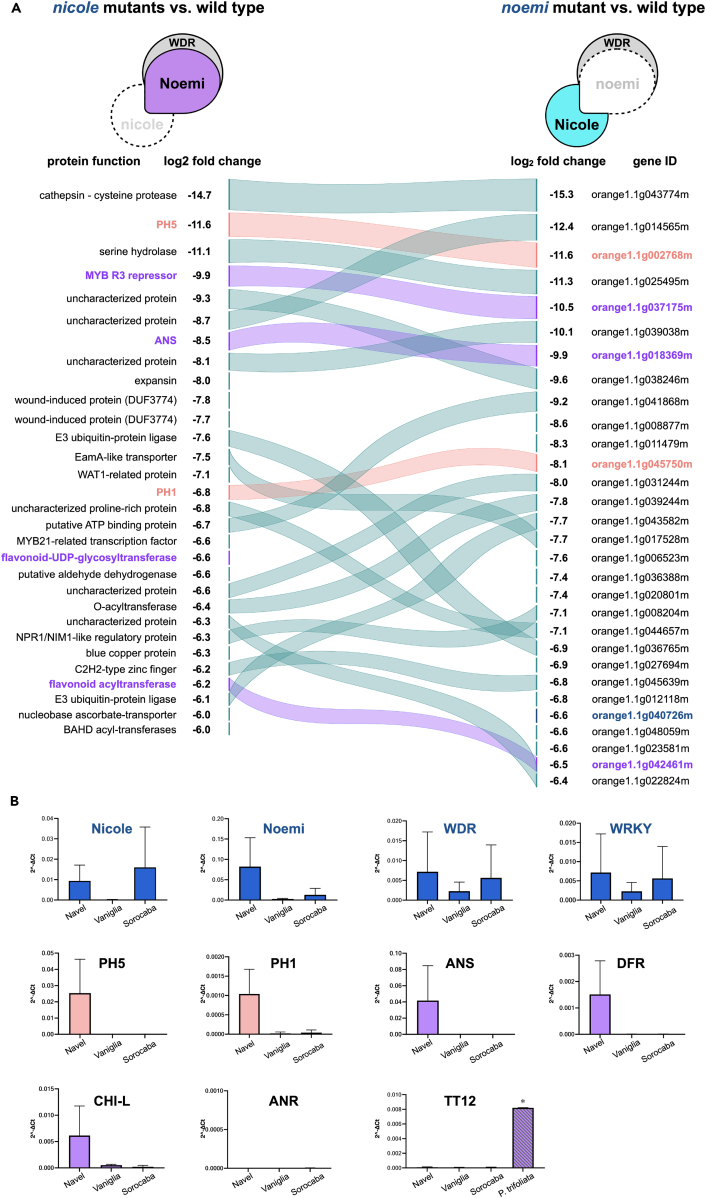
Nicole and Noemi regulate common processes and activate genes required for vacuolar hyperacidification and PA biosynthesis (A) Alluvial plot illustrating the relationships between genes downregulated in *nicole* (“Lima Verde” and “Lima Sorocaba”) and *noemi* (“Vaniglia”) mutants compared to wild type (“Navel”). Differentially expressed genes from RNA-seq data were ranked by fold change. Curved lines in color connect identical genes. *PH5* and *PH1* are indicated in light red; PA-related genes in purple. The top eight ranked genes are in common between the two sets of mutants. (B) Expression analysis of the regulatory genes encoding transcription factors forming the MBW complex and validation of changes observed in RNA-seq data for genes encoding the P-type ATPases PH5 and PH1and genes involved in PA biosynthesis. Note that *ANR* is not expressed in any of the sweet orange varieties analyzed. For *TT12*, the expression in *P. trifoliata* is shown for comparison. Gene expression was determined by RT-qPCR and normalized to the reference gene, actin. Error bars represent the standard deviation of the mean (*n* = 3 except for *P. trifoliata*, *n* = 2). Asterisks indicate comparative statistical significance relative to “Navel”: ∗ *p* < 0.01. See also Figure S3 and Table S1.

We then analyzed the transcriptome of the juice vesicles of fruit of three *nicole* mutants (one accession of “Lima Sorocaba” and two accessions of “Lima Verde” obtained from different sources) and compared these with “Navel” as a wild-type control for acidity, and with the *noemi* “acidless” mutant “Vaniglia”.^12^ We found that a total of 1,151 genes were downregulated (*p* value <0.05) in all three *nicole* mutants, 636 of which were shared by “Vaniglia”, indicating that Nicole and Noemi regulate common processes. Remarkably, the top eight downregulated genes were the same in the two sets of mutants (Figure 2A). Among them were *PH5*, whose expression was completely abolished in both *noemi* and *nicole* mutants and anthocyanin synthase (*ANS*), whose apparently paradoxical expression in fruit of wild-type orange had been reported previously.^26^ *ANS* is exclusively involved in the biosynthesis of anthocyanins and PAs (Figure S3A). Generally, anthocyanins are absent in citrus fruit^15^ which are also known to lack PAs and their monomers; they are listed as such by USDA, and considered safe for the tannin intolerant.^27^^,^^28^ Our analyses confirmed the absence of PAs in flesh and juice of sweet orange (Figures S3B–S3F).

Expression analysis by quantitative real-time reverse-transcription PCR (RT-qPCR) (Figure 2B) confirmed that neither of the genes encoding P-type ATPases, *PH5* and *PH1*, were expressed in the *nicole* (“Lima Sorocaba”) or *noemi* (“Vaniglia”) orange mutants, unlike in wild type “Navel”, providing conclusive evidence that fruit hyperacidification is controlled by the Nicole-Noemi complex. *ANS* displayed a similar behavior, and so did *DFR*, another structural gene exclusively involved in anthocyanin and PA biosynthesis. A chalcone isomerase-like gene (*CHI-L*) required for efficient accumulation of PAs in Arabidopsis^29^ was downregulated in both *nicole* and *noemi* mutants. Among other PA biosynthetic genes, we detected no expression of *LAR* and *ANR* in fruit of any orange varieties while a gene encoding a PA MATE transporter homologous to TT12^30^ was barely expressed in any of the genotypes examined.

**Figure 3:**
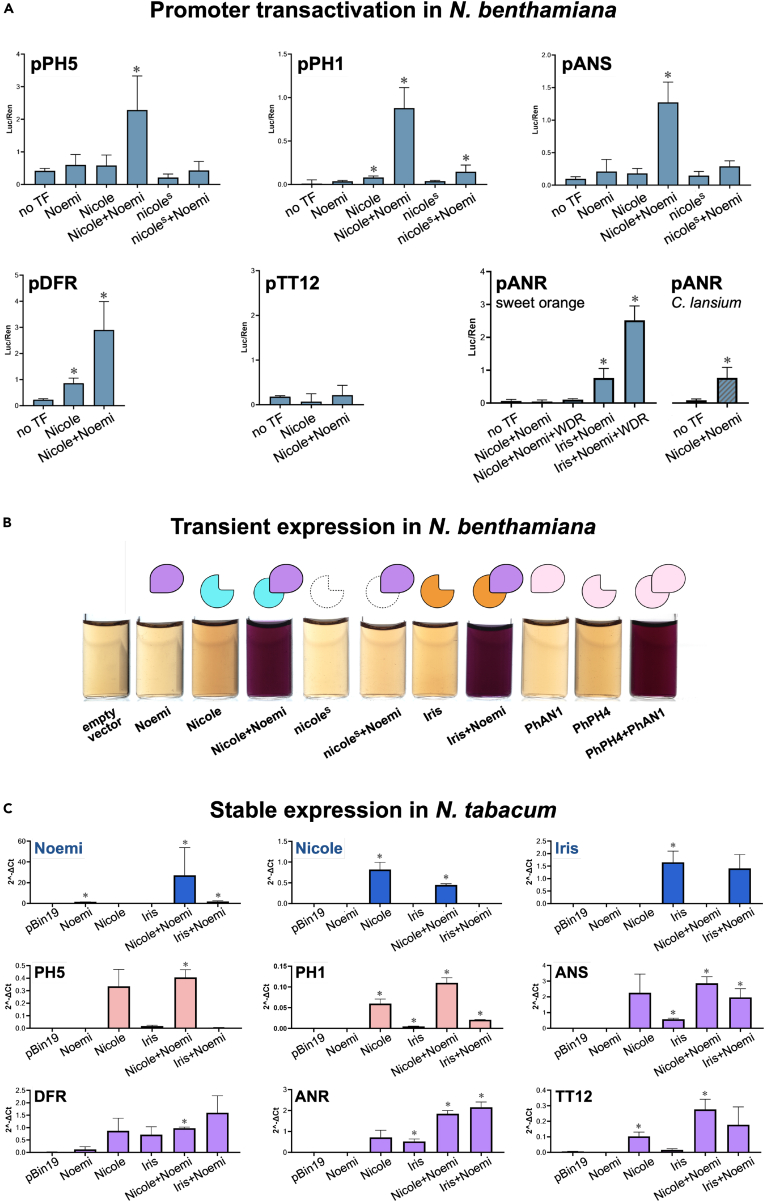
The Nicole-Noemi complex activates the citrus promoters of PA biosynthetic genes and induces PA production when expressed in tobacco (A) Transactivation assays using the Dual-Luciferase reporter system showing the ability of the Nicole-Noemi complex to activate the citrus promoters of *PH5*, *PH1*, and several PA biosynthetic genes. The mutated protein with a C-terminal deletion, nicole^S^, has little or no effect. Note the inability of Nicole-Noemi complex to activate *pANR* from sweet orange (both *pANR*^*1*^ and *pANR*^*2*^ tested) even in the presence of WDR. Error bars represent the standard deviation of the mean (*n* = 5). Asterisks indicate comparative statistical significance relative to “no TF (transcription factor)” control: ∗ *p* < 0.01. (B) Transient expression of *Nicole* and *Noemi* in *N. benthamiana* results in the accumulation of DMACA-positive metabolites; extracts of infected leaves were mixed mix 0.5 volumes of 0.3% DMACA reagent. A purple pigmentation indicates the production of PAs or their monomers. (C) Comparative analysis of tobacco plants transformed with the regulatory MYB genes *Nicole* or *Iris* with or without coexpression of the bHLH *Noemi.* Nicole can activate the P-type ATPases *PH5* and *PH1* and all the genes required for PA biosynthesis. Gene expression was determined by RT-qPCR and normalized to the reference gene, actin. Error bars represent the standard deviation of the mean (*n* = 3). Asterisks indicate comparative statistical significance relative to “pBin19” control: ∗ *p* < 0.01. See also Figure S3, Table S1 and Data S2.

Transient transactivation in *N. benthamiana* using a dual luciferase reporter assay demonstrated the ability of the Nicole-Noemi complex to activate the promoters of *PH5*, *PH1*, *ANS*, and *DFR* (Figure 3A). The transcription factors had no activity on the *ANR* promoter. In the same experiments, the truncated version of *Nicole (nicole*^*s*^*)*, represented by the coding sequence isolated from “Lima Sorocaba” juice, was only marginally active, confirming the effect of the transposable element insertion on the activity of *Nicole*. Iris and Noemi activated the *ANR* promoter of sweet orange (Figure 3A), indicating that the accumulation of PAs in the seed coat of sweet orange, including “Lima Sorocaba”and “Lima Verde” is controlled by the Iris-Noemi complex.

### Nicole induces proanthocyanidin accumulation when expressed in tobacco

During the transient assays we also tested the impact of Nicole and Noemi on acidification of leaf tissues, but we found no evidence of a change in pH (Figures S3G and S3H) although we did detect the production of significant amounts of PAs by staining crude extracts of leaf tissue around inoculation sites with DMACA (Figure 3B). In contrast, the defective *Nicole* allele was unable to induce the production of DMACA-positive metabolites after transient expression in *N. benthamiana*. Interestingly, leaves infiltrated with *PhPH4* and *PhAN1*, the known regulators of vacuolar acidity in petunia, produced the same DMACA-positive stain, which was also induced by the combination of Iris and Noemi (Figure 3B).

When stably expressed in *N. tabacum*, *Nicole* induced the production of PAs (Figure S3I) and, correspondingly, the expression of PA biosynthetic genes (Figure 3C). *PH5* and *PH1* were also strongly upregulated. There was little or no activity on the expression of two genes exclusively involved in anthocyanin biosynthesis, 3-GT and PAT (Figure S3A), suggesting that *Nicole* retains its specificity as a PA activator when expressed in tobacco.

Overall, our data in tobacco indicate that the Nicole-Noemi complex directly activates not only *PH1* and *PH5*, key genes required for vacuolar hyperacidification but also genes involved in PA biosynthesis. However, while the entire PA pathway is activated when *Nicole* and *Noemi* are overexpressed in tobacco, only a subset of genes are targets of the complex in sweet orange.

### *ANR*, a gene essential for proanthocyanidin biosynthesis, is not expressed in fruit of sweet orange

In sweet orange, three genes required for PA biosynthesis and accumulation are not expressed in wild-type fruit: *LAR*, *TT12*, and *ANR*. We hypothesized that, during citrus evolution, one of these genes became irresponsive to the Nicole-Noemi complex, favoring additional mutations to arise in other PA genes whose expression became unnecessary in fruit.

*LAR* is not essential for PA biosynthesis since it is absent in the genome of some species, such as Arabidopsis, which can still produce PAs, formed exclusively on epicatechin units. Furthermore, *LAR* is highly expressed in fruit of citron (*C. medica*) and dependent on the activity of *Noemi* as indicated by the lack of expression in a previously characterized, “acidless” variety^12^ (Figures S4A and S4B).

The expression of the transporter *TT12* is negligible in orange. However, we measured high expression in fruit of trifoliate orange (*Poncirus trifoliata*) (Figures 2B, S4C, and S4D), an important species sexually compatible with citrus and now classified as a pure species within the *Citrus* genus.^31^ Trifoliate orange is characterized by very acidic fruit.

*ANR* is the only PA biosynthetic gene that is not expressed in any of the citrus species tested. Unlike *LAR*, *ANR* has been argued to be essential for PA biosynthesis and mutations in this gene result in large reductions in seed coat PAs in Arabidopsis^32^ and Medicago.^33^ However, it remains unclear whether this is a universal feature in all plants that make PAs.^34^

**Figure 4:**
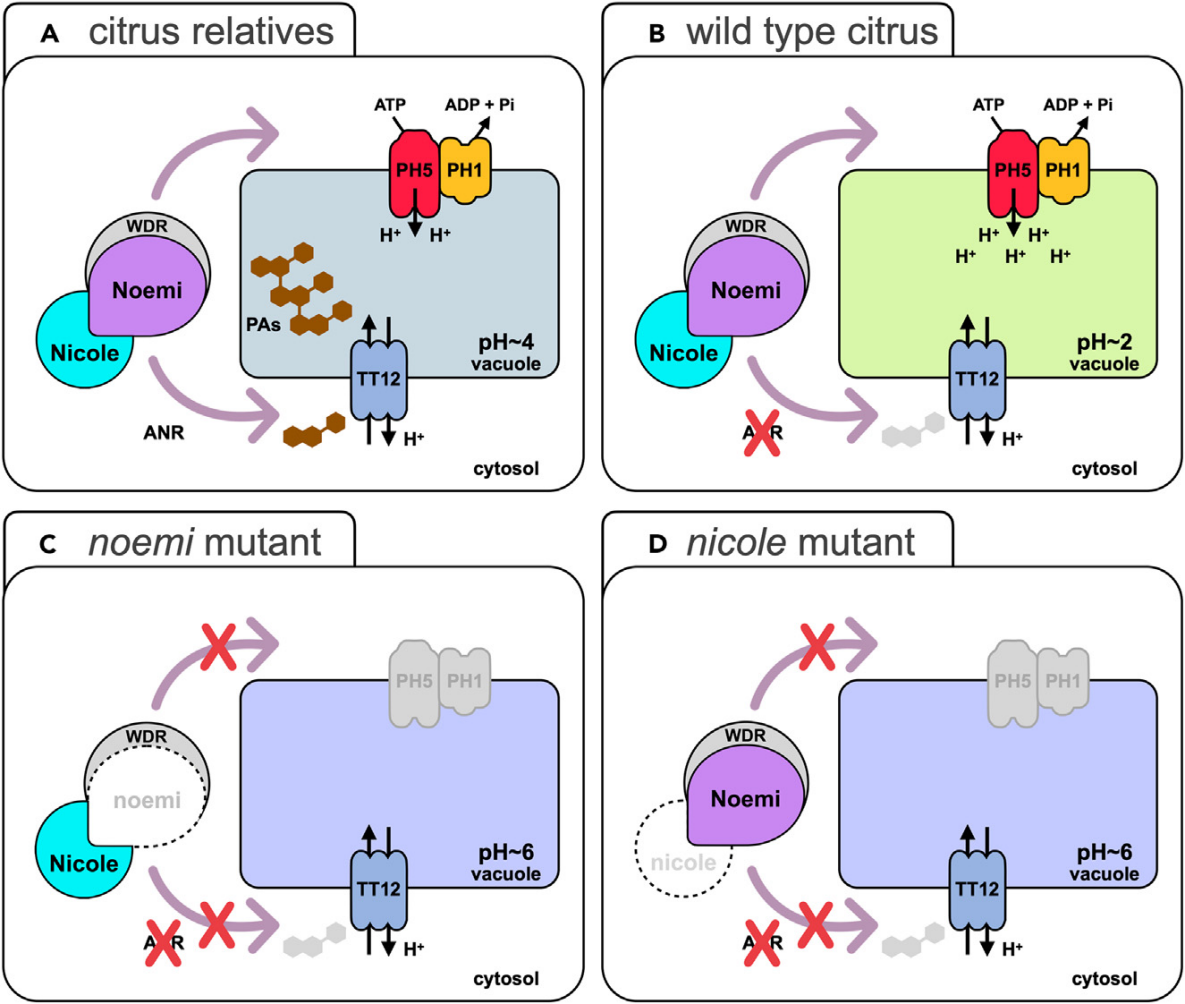
Simplified model of vacuolar hyperacidification in fruit of different citrus accessions and distant relatives (A) In distant relatives of the *Citrus* genus, the Nicole-Noemi complex activates the expression of PA biosynthetic genes and genes encoding the P-type ATPases PH1 and PH5, resulting in vacuolar hyper acidification required for uptake of PA monomers through the H^+^ antiporter TT12. (B) In citrus, loss of Nicole-Noemi activation of *ANR*, which is essential for PA biosynthesis, and low expression of other biosynthetic genes abolish PA production and generate extreme vacuolar hyper acidification which is not compensated by the import of PA monomers. (C and D) In citrus mutants *noemi* or *nicole*, the upregulation of the redundant PA biosynthetic genes and the genes encoding P-type ATPases is completely abolished, preventing any possible hyperacidification. In these mutants, strong reduction in vacuolar acidity prevents the movement of citrate into the vacuole down its concentration gradient. See also Figure S4.

We observed that mature seeds of trifoliate orange are DMACA negative (Figure S4E), and the *ANR* gene is disrupted in this species, where a region of 2.5 kb spanning the last five exons is replaced by a 2.7 kb insertion corresponding to a repetitive element (Figure S4F). This observation demonstrated that *ANR* is essential for PA biosynthesis in this species. These findings suggested that it was the loss of activity of *ANR* that was the key to the loss of PAs in fruit during citrus evolution. In an ancestral Rutaceae, Nicole may have lost the ability to activate *ANR*, while retaining its ability to activate other PA biosynthetic genes including *PH5* and *PH1*. We speculate that the consequent hyperacidification of the vacuole could not be balanced by the H^+^ antiporter activity of TT12, since this transporter is dependent on the production of glycosylated epicatechin PA monomers,^30^^,^^35^ for which *ANR* is essential^36^ (Figure 4).

### *ANR* is expressed in fruit of distant citrus relatives, which also accumulates proanthocyanidins

To test this hypothesis, we searched for species related to citrus, within the family Rutaceae, producing fruits which have retained a functional PA biosynthetic pathway and tested whether these had lower fruit acidity than citrus. The genus *Citrus* belongs to the subfamily Aurantioideae, which is composed of two tribes: Clauseneae and Citreae (to which *Citrus* belongs)^37^ (Figure S4G). We obtained fruits from species representative of both tribes, and we tested them for PA production after DMACA staining. As for *Citrus*, the three species within the Citreae tribe, *Atalantia ceylanica*, *Aegle marmelos*, and *Severinia buxifolia*, produced DMACA-negative fruit, but there was strong staining of fruit from the more distantly related members of the Clauseneae tribe: *Glycosmis pentaphylla*, *Clausena anisata*, and *Clausena lansium* (Figure S4H). Fruit of these species had pH in the range 3.7–5.5. We took advantage of the availability of the genome of *Clausena lansium*^38^ to isolate the sequence of the *ANR* gene and its promoter. The coding sequences shared 95% nucleotide identity with the two *ANR* alleles of orange but differed considerably in the size of the first intron and in their promoter regions (Data S2). In agreement with the detection of PAs, *ANR* was expressed in fruit of *Clausena lansium* in contrast to orange (Figure S4I) and, unlike orange, the promoter of *ANR* from *Clausena lansium* could be activated by the Nicole-Noemi complex (Figure 3A). We propose a model where *Citrus* may have lost the ability to produce PAs in fruit as a result of a change in the ability of Nicole to activate *ANR*. The inability to produce PAs and the retention of activation of the proton pumps on the tonoplast may have led to hyperacidification through the failure of the MATE proton antiporter to release the proton gradient in the absence of its cargo (Figure 4). This hyperacidification may have been reinforced by the low expression of *DFR* and by the loss of Nicole-Noemi activation of *TT12*, since the equivalent transcription factors in Arabidopsis, AtMYB5 and TT8 can activate *TT12* expression, as well as, at least weakly, activate *ANR*.^20^ The ability to produce PAs in fruit has been retained in distantly related species within the same Aurantinoideae subfamily and is associated with lower fruit acidity.

## Discussion

The identification and characterization of natural mutants of sweet orange has provided proof that *Nicole* encodes a key transcription factor controlling fruit acidity in citrus. Our study uncovered an extensive overlap between fruit acidity and PA biosynthesis.

In seeds, we have identified a different MYB transcription factor, Iris, controlling production of PAs. When overexpressed in tobacco, both *Nicole* and *Iris* promote PA accumulation through transcriptional activation of all the biosynthetic genes, but only Nicole strongly upregulates the genes encoding the P-type ATPases PH5 and PH1.

In general, the presence of PAs in seeds is almost a universal feature in higher plants and it does not strictly depend on the activity of PH5 and PH1. In fact, plants that do not have either of the corresponding genes in their genomes, for example tomato, are fully capable of producing seed PAs. Nevertheless, the co-regulation of genes required for vacuolar hyperacidification with those involved in PA biosynthesis is not surprising. In Arabidopsis (which does not have a functional PH1), the gene encoding the P-type ATPase equivalent to PH5, *AHA10*, is activated by TT2 during PA biosynthesis, as well as by AtMYB5, encoded by the ortholog of *Nicole*, which has been reported to regulate PA biosynthesis when expressed in the seed coat, through its ability to partially complement *tt2* mutants.^20^ This coregulation is plausible since the transport of PA precursors into the vacuole depends on the electrochemical gradient generated by PH5^35^^,^^39^^,^^40^ required to energize the H^+^ antiporter TT12. Considerable intersection between these two processes has been documented in other species^41^^,^^42^^,^^43^ including grape, where two homologs of Nicole activate, with different efficiency, partially overlapping sets of genes.^44^

In the plant kingdom, the presence of PAs in tissues other than seeds is less common and, according to the analysis of the distribution of *PH1* and *PH5* among plant species,^45^ appears to be strictly associated with the presence of both ATPases. Fruit of many plants as diverse as apple (dicot) and banana (monocot) are known for their PA content and so the receptacle of strawberry and the leaves of some legumes. All these plant species contains both *PH1* and *PH5*, causing vacuolar hyperacification which may be required not only for the transport of PA monomers across the tonoplast but also for their condensation into polymeric PAs, the final and yet poorly understood step in PA biosynthesis.^34^

Citrus fruit, despite having a functional PA pathway, *PH1*, *PH5*, and expressing *Nicole*, the essential regulator of both sets of genes, do not produce fruit PAs. We associated this feature with the inability to activate *ANR*, the only PA biosynthetic gene for which we could not detect expression in any of the citrus fruit analyzed. The activation of *ANR* by the Nicole-Noemi complex has been retained in fruit of distant citrus relative which, accordingly, accumulate PAs and are also characterized by lower fruit acidity compared to citrus.

We speculate that, in citrus, loss of ANR activity in fruit juice vesicles results in vacuole hyperacidification that is not counteracted by the import of PA precursors. Since plant vacuolar pH is tightly homeostatically regulated, other mechanisms must be in place to ensure that the very steep electrochemical H^+^ gradient across the tonoplast is maintained during fruit ripening. Our study, however, indicates that loss of induction of PA biosynthesis may be an integral component of the hyperacidification mechanism in citrus fruit.

### Limitations of the study

We propose an evolutionary scenario where the molecular mechanism required for the extreme hyperacidification in citrus fruit may have been co-opted from the proanthocyanin biosynthetic pathway. Although the results presented are consistent with this hypothesis, we do not have direct evidence to support our model (Figure 4B). In particular, we could not establish that lack of ANR activity prevents the efflux of protons induced for PA antiport into the vacuoles of juice sacs, resulting in their hyperacidification. The generation of a knockout *anr* mutant in *N. benthamiana* followed by overexpression of *Nicole* and *Noemi* provided inconclusive results because of a failure of the mutation to inhibit PA accumulation. Further attempts to measure the vacuolar pH in the cells of the inner seed coat of an *anr* mutant of Arabidopsis were unsuccessful because the pH-sensitive fluorescent dye used was unable to permeate this cell layer.

## Resource availability

### Lead contact

Further information and requests for resources should be directed to and will be fulfilled by the lead contact Eugenio Butelli: eugenio.butelli@jic.ac.uk.

### Materials availability

DNA constructs and seeds of transgenic plants generated in this study are available from the lead contact upon request.

### Data and code availability

• DNA sequences and RNA-seq reads were deposited into NCBI as indicated in “key resources table”.
• All data reported and additional information required to reanalyze the data reported in this paper are available from the lead contact upon request.
• This paper does not report original code.

## Acknowledgments

E.B. and C.M. were supported by the Institute Strategic Programmes: ‘‘Harnessing Biosynthesis for Sustainable Food and Health-HBio’’ (BB/X01097X/1) and ‘‘Molecules from Nature’’ (BB/P012523/1) from the Biotechnological and Biological Scientific Research Council (10.13039/501100000268BBSRC, United Kingdom). E.A. was supported by 10.13039/501100000268BBSRC iCASE studentship “Understanding the molecular determination of quality traits in citrus fruit” (MARTIN_J17ICASE). We thank Ingo Appelhagen for guidance on 6-CFDA staining to measure pH of cells, Alan Houghton for advice on designing Figure 2, and Phil Robinson for photography.

## Author contributions

E.B. and C.M. planned and designed the research; E.B., E.A., E.S., C.C, E.W., L.H., and C.C.H. performed experiments; L.P. C.L., and A.G.-L. provided plant material and information; E.B. and C.M. wrote the article with input and comments from the other authors.

## Declaration of interests

The authors declare no competing interests.

## STAR★Methods

### Key resources table

**Table:** 

REAGENT or RESOURCE	SOURCE	IDENTIFIER
**Bacterial and virus strains**		

*A. tumefaciens* LBA4404	Lab stock	N/A
*A. tumefaciens* GV3101	Lab stock	N/A

**Chemicals, peptides, and recombinant proteins**		

4-Dimethylaminocinnamaldehyde (DMACA)	Sigma-Aldrich	Cat # D4506
(+)-Catechin	Sigma-Aldrich	Cat # 43412-10MG
(−)-epicatechin	Sigma-Aldrich	Cat # 68097-10MG
6-carboxyfluorescein diacetate (6-CFDA)	Sigma-Aldrich	Cat # C5041

**Critical commercial assays**		

DNeasy Plant Mini Kit	QIAGEN	Cat # 69106
RNeasy Plant Mini Kit	QIAGEN	Cat # 74904
Phusion High-Fidelity DNA Polymerase	Thermo Fisher	Cat # F530L
SuperScript IV Reverse Transcriptase	Thermo Fisher	Cat # 18090050
SYBR Green JumpStart Taq ReadyMix Kit	Sigma-Aldrich	Cat # S4438-500RXN
Dual-Glo Luciferase Assay System	Promega	Cat # E2920
Custom DNA sequencing	Eurofins	https://www.eurofinsgenomics.eu/

**Deposited data**		

Nucleotide sequence of wild type *Nicole* in sweet orange and downstream T/G polymorphism	This study	GenBank: OR_766345
Nucleotide sequence of mutated *nicole* in sweet orange “Lima Verde” and “Lima Sorocaba”	This study	GenBank: OR_766346
RNA-Seq data deposited into NCBI sequence read archive (SRA)	This study	NIH BioProject: PRJNA1071547

**Experimental models: Organisms/strains**		

Accession of sweet orange (*C. sinensis*)	Listed in ‘Plant Material’	N/A

**Oligonucleotides**		

See Table S2 for primers	This study	N/A

**Recombinant DNA**		

pDONR 207	Thermo Fisher	N/A
pJAM1502	This study	N/A
pEAQ-HT-DEST1	Sainsbury et al.^46^	N/A
pGreen II 0800-LUC	Hellens et al.^47^	N/A

**Software and algorithms**		

PhyML	Guindon et al.^48^	http://www.atgc-montpellier.fr/phyml/binaries.php
RAxML	Stamatakis et al.^49^	https://cme.h-its.org/exelixis/web/software/raxml/
topGO	Alexa et al.^50^	https://bioconductor.org/packages/release/bioc/html/topGO.html
DEGUST	Powel et al.^51^	http://degust.erc.monash.edu/

**Other**		

Citrus Genome Assembly and Annotation	*Citrus sinensis* v1.1	https://phytozome-next.jgi.doe.gov/info/Csinensis_v1_1
Citrus Genome Assembly and Annotation	CPBD: Citrus Pan-genome	http://citrus.hzau.edu.cn/
Citrus Genome Assembly and Annotation	*Citrus sinensis* “Valencia” (set A)^25^	NIH BioProject: PRJNA736174
Citrus Genome Assembly and Annotation	*Citrus sinensis* “Valencia” (set B)^25^	NIH BioProject: PRJNA736176

### Experimental model and study participant details

#### Plant material and growth conditions

Accessions of sweet orange and citrus relatives were obtained from different sources as indicated in ‘method details’. Transgenic tobacco plants were obtained in the Samsun NN line of *Nicotiana tabacum* and kept in greenhouse conditions. Greenhouses were kept to 20^o^C in the day and 16^o^C at night. Supplemental lighting was used in winter months, maintaining a 16-h day.

### Method details

#### Plant material

“Navel” and “Valencia” are acidic sweet orange varieties, wild type for acidity used as comparative controls. For RNA-seq analysis, ripe fruit of one “Navel” accession were obtained from CREA (Acireale, Italy). The other biological replicates of “Navel” oranges were obtained from a local supermarket in Norwich (Tesco). “Valencia” fruits were obtained from a local supermarket in Norwich (Waitrose). “Vaniglia” fruits for all the biological replicates were obtained from CREA. “Lima Sorocaba” was obtained from a local market in São Paulo region, Brazil. “Lima Verde” fruits were obtained from Santa Cruz do Rio Pardo (R1) and Mogi Guaçu (R2). Fruits of *Severinia buxifolia* were obtained from CREA. The remaining distant citrus relatives were obtained from Instituto Valenciano de Investigaciones Agrarias (IVIA, Valencia, Spain). *P. trifoliata* plant material was obtained from a tree in Norwich, grown from seeds collected at CREA.

#### pH and citric acid analyses

Fruit juice was obtained using a conventional citrus squeezer from fruits of sweet orange and *P. trifoliata* and with a plastic pestle from fruit of distant citrus relatives. After centrifugation, pH of juice was measured using a standard combination Ag/AgCl pH electrode in at least three fruits per variety. Citric acid in different sweet orange varieties was quantified by gas chromatography-mass spectrometry (GCMS) as described by Lin et al.^52^ Three biological replicates, obtained from individual fruits, were used except for “Navel” where only two samples were available.

#### Isolation of *Nicole* alleles

Full-length *Nicole* alleles were isolated by PCR using primers PH4-FA and PH4-RZ (Table S2) designed close to start and stop codons of the gene. DNA was isolated from leaves or fruit of different sweet orange varieties using the DNeasy Plant Mini Kit (Qiagen). PCR fragments were used directly for sequencing. Appropriate primers were used to obtain the complete sequences and confirm the presence of the retrotransposon. All the sequences obtained were compared to those available in different citrus genome assemblies listed in the key resources table.

#### Phylogenetic analysis of citrus MYB transcription factors

Genome-wide analyses of R2R3-MYB proteins in the sweet orange v1.1 Phytozome reference genome were performed by protein sequence analyses, multiple sequence alignments, and subsequent phylogenetic tree construction. Other proteins from different species were also included: 125 R2R3-MYBs from Arabidopsis and characterised R2R3-MYBs from *V. vinifera* (*n* = 5), *P. hybrida* (*n* = 2), *L. chinensis* (*n* = 2), *M. truncatula* (*n* = 3), *M. domestica* (*n* = 4), *P. persica* (*n* = 3), *G. max* (*n* = 1) and *F. ananassa* (*n* = 1). Initially, candidate genes encoding MYB TFs were identified by reference genome amino acid sequence analysis of PFAM domains PF00249 (Myb-like DNA binding domain). Candidate sweet orange MYB-like TFs containing two predicted MYB domains were selected and designated as R2R3-CsMYBs (Table S1). The maximum-likelihood (ML) substitution model that best described each alignment was determined using PhyML.^48^ Phylogenetic trees were constructed using the best substitution model with RAxML^49^ (1,000 bootstrap replicates).

#### RNA extraction from sweet orange fruit and sequencing

RNA from sweet orange fruits of different varieties was extracted as previously reported^53^ and further purified using an RNeasy Plant Mini Kit (Qiagen). RNA samples were sent to Novogene for low input PE150 Illumina sequencing. Novogene conducted the initial analyses, providing FPKM values for all genes in all samples and significance values when compared to expression in wild type “Navel” fruit juice. R (v4.0.5) was used to generate lists of differentially expressed genes in all the mutant varieties and GO enrichment analyses using R package topGO.^50^ For comparative differential expression analyses between wild type and ‘acidless’ *noemi* mutant or the combined three *nicole* mutants, read counts were analyzed using the interactive web-tool DEGUST using the Voom function from limma package methodo methodology.^51^

#### Real-time quantitative PCR analysis

Quantitative real-time reverse-transcription PCR (RT-qPCR) was used to validate RNA-seq transcriptomic data and to analyze gene expression in transgenic tobacco. For sweet orange, total RNA was extracted as described above, treated with DNAse I (Roche) and retrotranscribed using Superscript IV reverse transcriptase (Invitrogen). RT-qPCR primers were designed to span exon-exon junctions or were separated by large introns to avoid amplification of residual DNA contamination and tested for primer efficiency. Reactions were conducted using the SYBR Green JumpStart Taq ReadyMix Kit (Sigma) with the X96 Touch Real-Time PCR Detection System (Biorad). Three biological and three technical replicates were used for each variety of sweet orange. For conventional RT-PCR, first-strand cDNA was obtained as described above and amplified with Phusion High-Fidelity DNA Polymerase (Thermo Fisher) and primers indicated in Table S2. DNA bands were sequenced to confirm correct exon-exon junctions.

#### Transient expression in *N. Benthamiana* and dual luciferase assay

The effect of different transcription factors on PA production and promoter activation was assessed by transient expression via agroinfiltration of leaves of *N. benthamiana*. Effector plasmids encoding transcription factors from sweet orange were obtained by PCR amplification using cDNA from fruit of sweet orange or petals of petunia (V26) and primers listed in Table S2. The coding sequences were first cloned into the pDONR207 entry clone (Thermo Fisher) and subsequently transferred to the destination vector pEAQ-HT-DEST1^46^ designed for Gateway cloning technology (Invitrogen). The reporter constructs were obtained by PCR amplification of different promoters followed by restriction enzyme cloning into the pGreen II 0800-LUC vector^47^ to control the expression of the firefly-derived luciferase reporter gene. This vector also contained a *Renilla* luciferase gene under the control of a *CaMV 35S* promoter as an internal control to normalise the values of the experimental reporter gene. Effector and reporter plasmids were transformed into *A. tumefaciens* strain GV3101. Reporter plasmids were co-transformed with the helper plasmid pSoup. Liquid cultures were grown overnight with appropriate antibiotic selection and harvested by centrifugation. Cells were washed and resuspended in 10 mM MgCl_2_, 10 mM MES pH 5.6, 200 μM acetosyringone to A_600_ = 0.1 for effector and A_600_ = 0.2 for reporter constructs. *A. tumefaciens* suspensions were infiltrated into the abaxial surface of expanded leaves of *N. benthamiana*. For each combination, five injected areas were treated as biological replicates. Agroinfiltrated leaves were harvested after 3 days, and luciferase activity was measured immediately using the Dual-Glo Luciferase Assay System kit (Promega). Leaf discs of 4 mm in diameter were collected in 1.5 mL white transparent tubes containing 100 μL of PBS. A volume of 75 μL of luciferase assay reagent was added and firefly luminescence was measured on a Glomax 20/20 single tube luminometer (Promega) after 10 min. *Renilla* luminescence was measured on the same instrument 10 min after the addition to the same sample of 75 μL of Stop & Glo reagent. Results were expressed as the ratio of firefly to *Renilla* luciferase activity (Luc/Ren).

#### pH imaging in *N. Benthamiana*

The method employed a non-fluorescent dye, 6-carboxyfluorescein diacetate (6-CFDA, Sigma-Aldrich) which is cleaved by non-specific vacuolar esterases releasing the fluorescent dye 6-carboxyfluorescein (6-CF), which has a pH-dependent ratio of emission intensities at different excitation wavelengths. Two days after agroinfiltration, leaf discs were infiltrated with pH staining solution (20 μM 6-CFDA in MS liquid medium). Leaf discs were collected after 24 h and incubated with 13.3 μM 6-carboxyfluorescein di acetate (6-CFDA), 0.3% Triton X-100 in MS liquid medium. Images were captured using a Leica SP5 Confocal Stereo Microscope equipped with an HC PL APO CS2 20x/0.75 air objective (Leica Microsystems GmbH). The absorbance emission derived from 458 nm to 488 nm excitation lasers was measured and the 488/458 nm emission ratio was calculated and compared with a calibration curve obtained with 6-CF dye in buffers with different known pH.

#### Stable transformation in *N. tabacum* and expression analysis

The coding sequences of different regulatory genes were obtained as described above and cloned into pJAM 1502, a pBin19-derived binary vector equipped with a double *CaMV 35S* promoter and a *CaMV* terminator with *attR* recombination sites in between, using Gateway cloning technology. The resulting plasmids were transferred to A. tumefaciens strain LBA4404 and used to transform N. tabacum cv Samsun NN. Plants transformed with the empty pJAM 1502 were used as a control. For gene expression analysis, total RNA was extracted using an optimised cetyltrimethylammonium bromide (CTAB)-based protocol^54^ to minimise the binding of PAs to RNA. Sepals were used for the lines expressing individual regulatory genes and leaves for co-expressing lines, since *35S::Nicole* - *35S::Noemi* plants did not produce any flowers. RT-qPCR was conducted as described above. Three biological and three technical replicates were used for each transgenic plant. For the biological replicates, at least two independent transformants or, for the double overexpressors, two independent crosses between different transformants were used for each genotype.

#### Localisation and quantification of proantocyanidins

The presence of proanthocyanidins in seeds, fruit, leaves, and flowers was determined by staining with 0.3% (w/v) DMACA (4-Dimethylaminocinnamaldehyde, Sigma) in methanol and 6 M HCl (1:1, v/v) followed by several washing steps with 70% ethanol. For *N. tabacum* leaves and flowers, samples were first destained overnight in a solution of ethanol-glacial acetic acid (3:1, v/v). For transient expression in *N. benthamiana*, freeze-dried leaf samples were extracted with 80% methanol overnight and 0.5 vol. of 0.3% DMACA reagent was added. In presence of PAs or their monomers, the solution initially quickly turned blue-green, then purple. If the compounds were not present, the solution remained yellow. For HPLC analysis, PAs were extracted from fruit of different sweet orange varieties and, as controls, from grape skin and transgenic *N. tabacum* plants generated in this study. Samples were extracted twice with 10 vol. of an extraction solution, containing 70% (v/v) acetone and 0.5% (v/v) acetic acid, and sonication at room temperature for 30 min. Extractions were centrifugated at 8,000 rpm for 10 min and supernatants containing soluble PAs were pooled. Soluble PA samples were washed three times with chloroform, and a further three times with hexane, before freeze-drying overnight. PA powder was resuspended in 1 mL extraction solution per 1 g of fresh starting material. The extracts were diluted 5-fold in H_2_O and run on a Shimadzu Nexera UHPLC with Prominence diode array detector (UV/vis absorbance) and a 2020 single quad mass spectrometer. Separation was on a 50 × 2.1 mm, 2.6 μm particle size Kinetex EVO C18 column (Phenomenex) using a gradient of acetonitrile versus 0.1% (w/v) formic acid in H_2_O, run at 0.6 mL min^−1^ at 40°C. Detection was by UV absorbance collecting full spectra from 200 to 600 nm at 12.5 Hz with a time constant of 0.08 s, and by positive mode electrospray MS. The mass spec collected full spectra from *m/z* 100–900 in 0.1 s and monitored *m/z* 291(+) by single-ion-monitoring for 50 msec. Spray chamber conditions were 250°C desorbation line, 200°C heat block, 1.5 L min^−1^ nebulizer gas, and 15 L min^−1^ drying gas. The injection volume was 5 μL. Flavan-3-ol monomers were quantified at 279 nm by calibration against (+)-catechin and (−)-epicatechin standard curves. Following acid-catalysis of soluble PA extracts, flavan-3-ol terminal subunits from PA polymers were released in addition to extension subunits. Terminal subunit concentration was determined by the subtraction of free flavan-3-ol monomer concentration in uncleaved PA samples from the total flavan-3-ol monomer concentration in the acid-catalysed samples.

### Quantification and statistical analysis

Bar plots were generated using GraphPad Prism 10 and Excel version 2308. Error bars represent standard deviation of the mean (SD). Asterisks indicate comparative statistical significance relative to “Navel“ calculated by unpaired Student’s t test.

The number of the replicates assayed is described in the STAR Methods and in the figure legends.

## Supplemental information

Document S1. Figures S1‒S4, Data S1 and S2, and Tables S1 and S2
