# Supplementary material for: Distinctive acidity in citrus fruit is linked to loss of proanthocyanidin biosynthesis
Source: iScience. 2024 Sep 13;27(10):110923. doi: 10.1016/j.isci.2024.110923 (PMC11467675; doi:10.1016/j.isci.2024.110923)
Supplement: Document S1. Figures S1‒S4, Data S1 and S2, and Tables S1 and S2 [file mmc1.pdf]

iScience, Volume *27*

Supplemental information

Distinctive acidity in citrus fruit is linked

to loss of proanthocyanidin biosynthesis

Elliott Atkins, Emanuele Scialò, Chiara Catalano, Carmen Caballero Hernández, Eva Wegel, Lionel Hill, Concetta Licciardello, Leandro Peña, Andrés Garcia-Lor, Cathie Martin, and Eugenio Butelli

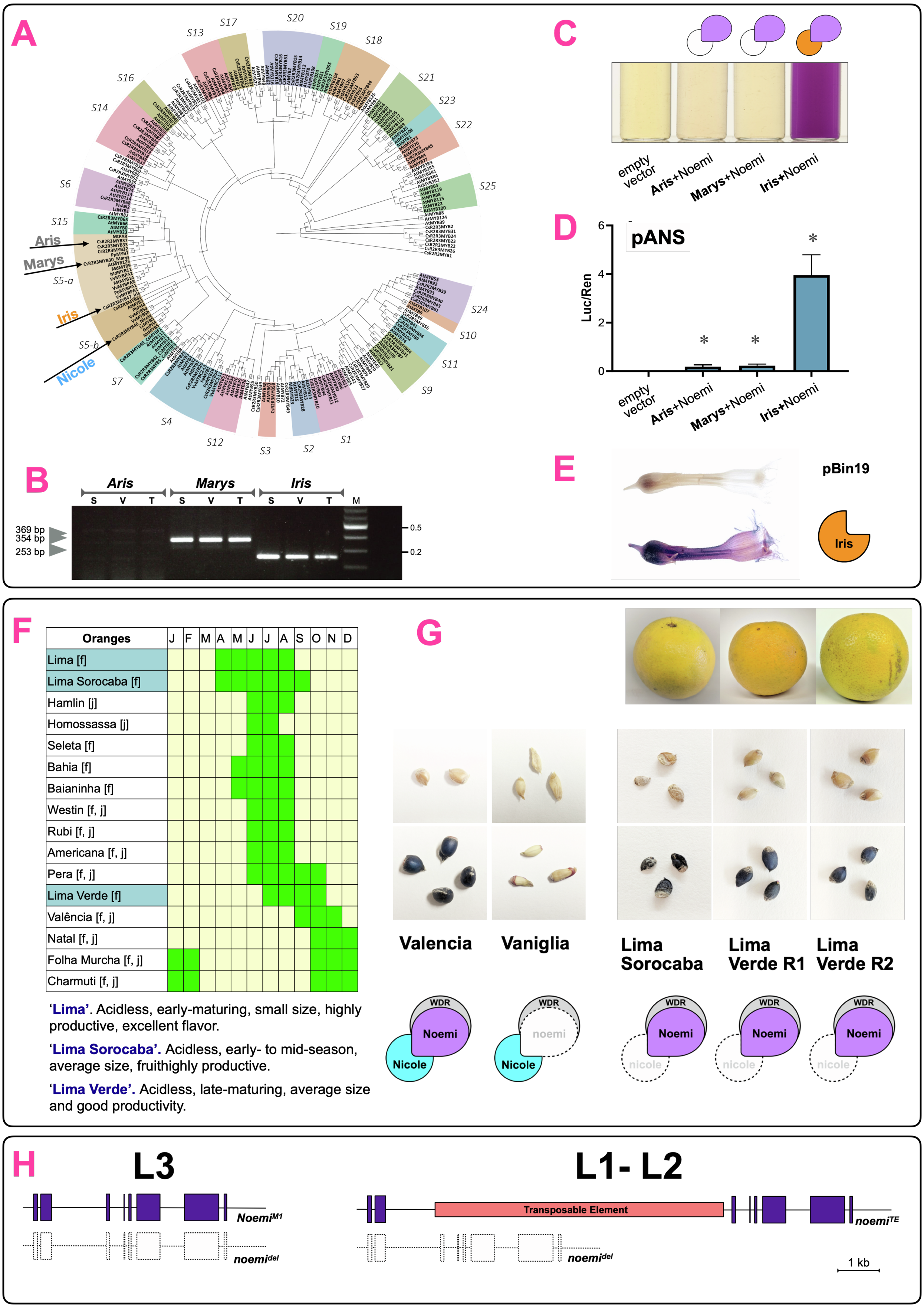
A
S13
S17
S20
S19
S18
S16
S21
S23
S14
S22
S6
S25
S15
Aris
Marys
S5-a
Iris
S24
S10
S5-b
Nicole
S11
S7
S9
S4
S12
S3
S2
S1
B
Aris
Marys
Iris
S V T S V T S V T M
369 bp
354 bp
253 bp
0.5
0.2
C
empty vector
Aris+Noemi
Marys+Noemi
Iris+Noemi
D
pANS
Luc/Ren
6
4
2
0
*
*
*
empty vector
Aris+Noemi
Marys+Noemi
Iris+Noemi
E
pBin19
Iris
F
Oranges J F M A M J J A S O N D
Lima [f]
Lima Sorocaba [f]
Hamlin [j]
Homossassa [j]
Seleta [f]
Bahia [f]
Baianinha [f]
Westin [f, j]
Rubi [f, j]
Americana [f, j]
Pera [f, j]
Lima Verde [f]
Valência [f, j]
Natal [f, j]
Folha Murcha [f, j]
Charmuti [f, j]
'Lima'. Acidless, early-maturing, small size, highly productive, excellent flavor.
'Lima Sorocaba'. Acidless, early- to mid-season, average size, fruithighly productive.
'Lima Verde'. Acidless, late-maturing, average size and good productivity.
G
Valencia
Vaniglia
Lima Sorocaba
Lima Verde R1
Lima Verde R2
WDR
Noemi
Nicole
noemi
nicole
H
L3
L1- L2
Transposable Element
Noemi^M1
noemi^del
noemi^TE
1 kb

Figure S1.

Figure S1. Phylogenetic analysis of R2R3-MYB transcription factors and 'Lima' varieties investigated in this study, related to Figure 1.

(A) Maximum-likelihood amino acid phylogeny of 68 putative CsR2R3-MYBs, 125 Arabiopsis R2R3-MYBs and 21 characterised R2R3-MYBs from other plant species. Subgroup (S1-25) designation conforms to the previously established phylogenetic characterisation of the Arabidopsis R2R3-MYB transcription factor family. Substitution model: JTT+G+F, bootstrap replicates = 1000. Node values are bootstrap value percentages. (B) RT-PCR analysis of cDNA isolated from the inner seed coat of three different varieties of sweet orange (S, 'Lima Sorocaba'; V, 'Lima Verde'; T, 'Tarocco') showing the expression of *Aris*, *Marys*, and *Iris*. M; 100 bp DNA ladder (NEB) (C) Transient expression of *Noemi* in combination with *Aris*, *Marys*, or *Iris* in *N. benthamiana*; extracts of infected leaves were mixed mix 0.5 volumes of 0.3 % DMACA reagent. A purple pigmentation indicates the production of PAs or their monomers. (D) Transactivation assays using the Dual-Luciferase reporter system showing the effect of *Noemi* in combination with *Aris*, *Marys*, or *Iris* on the promoters of *ANS*. Error bars represent the standard deviation of the mean (n=5). Asterisks indicate statistical significance relative to 'no transcription factor' control: $* \ p < 0.01$. (E) Flowers of control (pBin19) or transgenic tobacco plants constitutively expressing *Iris* under the control of the *CaMV 35S* promoter. Flowers were destained overnight in a solution of ethanol-glacial acetic acid (3:1, v/v) and stained with 0.3% DMACA in methanol-6M HCl (1:1, v/v) to show the presence of PAs. (F) Table showing time of maturity of the main commercial varieties of sweet orange in São Paulo state, Brazil. Letters between parentheses indicate predominant utilisation for fresh fruit market [f] and juice industry [j]. A brief description of fruit qualities of the three 'Lima' accessions considered in this study (highlighted in light blue) is provided. Table and information obtained from: http://www.citrolima.com.br/bulletin/bulletin7.htm. (G) Ripe fruits of 'Lima Sorocaba' and 'Lima Verde' obtained from different sources in São Paulo state, Brazil, immediately before sampling for pH, sugars, and organic acid content. Seeds from the same fruits were stained with DMACA reagent, highlighting the presence of proantocyanidins (PAs), unlike seeds of 'Vaniglia', a typical 'acidless' *noemi* mutant. A schematic diagram indicating if the orange varieties are wild type, *noemi*, or *nicole* mutants is also presented. (H) Allelic constitution of *Noemi* in 'Lima' (a *noemi* mutant distinct from 'Lima Verde' and 'Lima Sorocaba') determined by PCR analysis of genomic DNA extracted from leaves or seeds. In periclinal chimeras, cells of one meristematic layer are genetically different from the others. In citrus fruits, the juice vesicles are derived from L1 meristematic layer, where one allele of *Noemi* is deleted while the other contains a transposable element within the second intron [13]; seeds and segment walls are developed from L2; vascular bundles are produced by L3, where a functional *Noemi* allele is present.

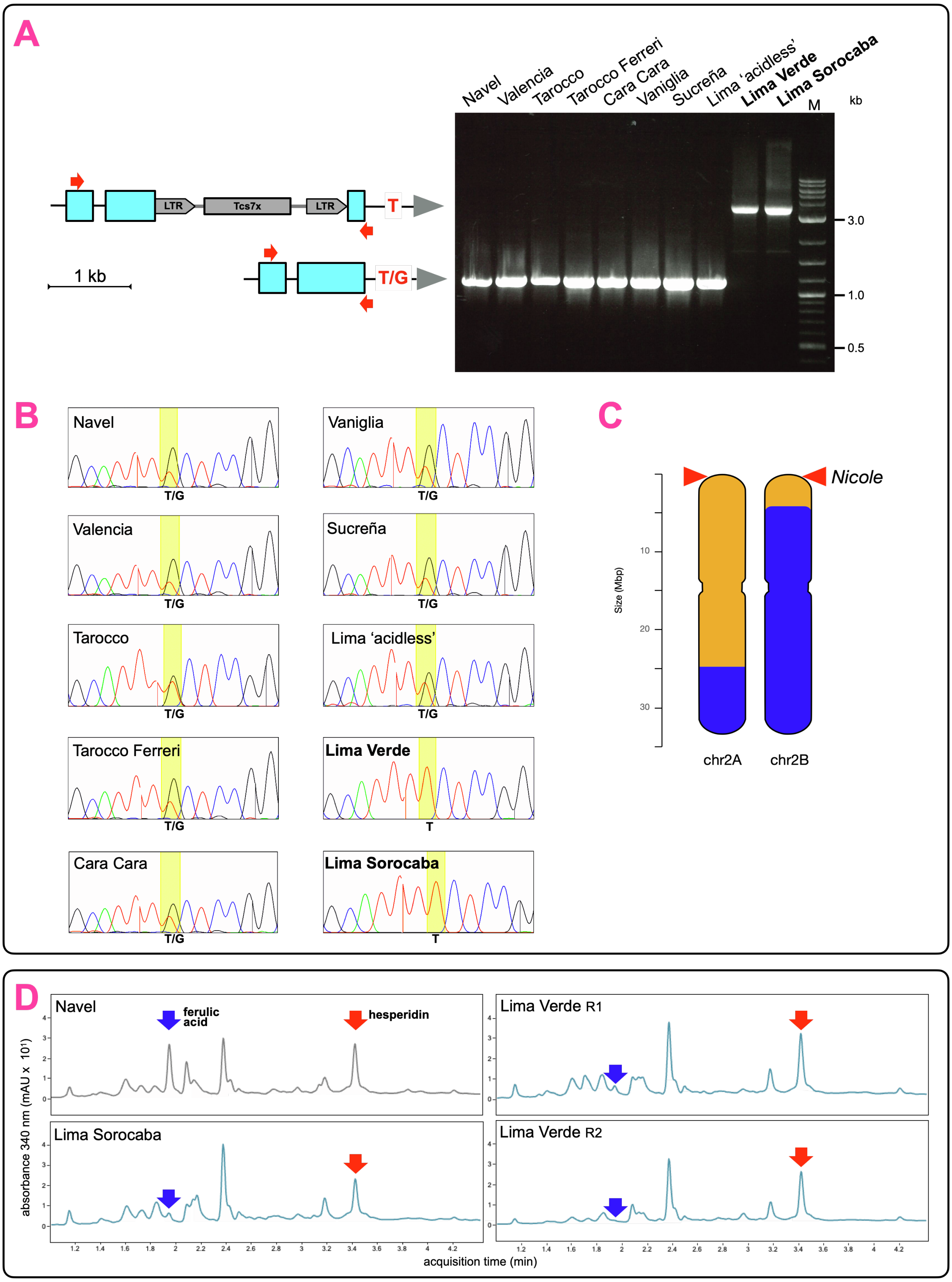
A
Navel
Valencia
Tarocco
Tarocco Ferreri
Cara Cara
Vaniglia
Sucreña
Lima 'acidless'
Lima Verde
Lima Sorocaba
M
kb
LTR
Tcs7x
LTR
T
T/G
1 kb
3.0
1.0
0.5
B
Navel
Valencia
Tarocco
Tarocco Ferreri
Cara Cara
Vaniglia
Sucreña
Lima 'acidless'
Lima Verde
Lima Sorocaba
T/G
T
C
Nicole
Size (Mbp)
10
20
30
chr2A
chr2B
D
Navel
ferulic acid
hesperidin
Lima Verde R1
Lima Sorocaba
Lima Verde R2
absorbance 340 nm (mAU x 10¹)
acquisition time (min)

Figure S2.

Figure S2. Genomic characterisation of *Nicole* in different varieties of sweet orange, related to Figure 1.

(A) Schematic diagram of *Nicole* determined by PCR analysis of genomic DNA extracted from leaves of different varieties. In 'Lima Verde' and 'Lima Sorocaba', *Nicole* is interrupted by a retrotransposon (Tcs7x) and no wild type alleles are present. Turquoise boxes represent exons; the retrotransposon is indicated in grey; red arrows indicate the approximate localization of the primers used for genomic PCR; M; 1 kb plus DNA ladder (NEB). (B) Chromatograms of the region 785 bp downstream the stop codon of *Nicole* highlighting the absence of a T/G polymorphism in 'Lima Verde' and 'Lima Sorocaba', indicating that one allele of *Nicole* is deleted. (C) Diagram showing the localisation of *Nicole* (red arrows) close to the telomeric region of chromosome 2. Orange and blue colours indicate the chromosome regions of mandarin and pummelo origin respectively [23]. (D) Chromatograms recorded at 340 nm, obtained by UHPLC, showing the detection of flavonoids and phenolic acids in juice of different varieties of sweet orange. The red arrows indicate hesperidin (hesperetin-7-O-rutinoside), the most abundant flavonoid in sweet orange; the blue arrows indicate ferulic acid, which is strongly reduced in all the *nicole* mutants.

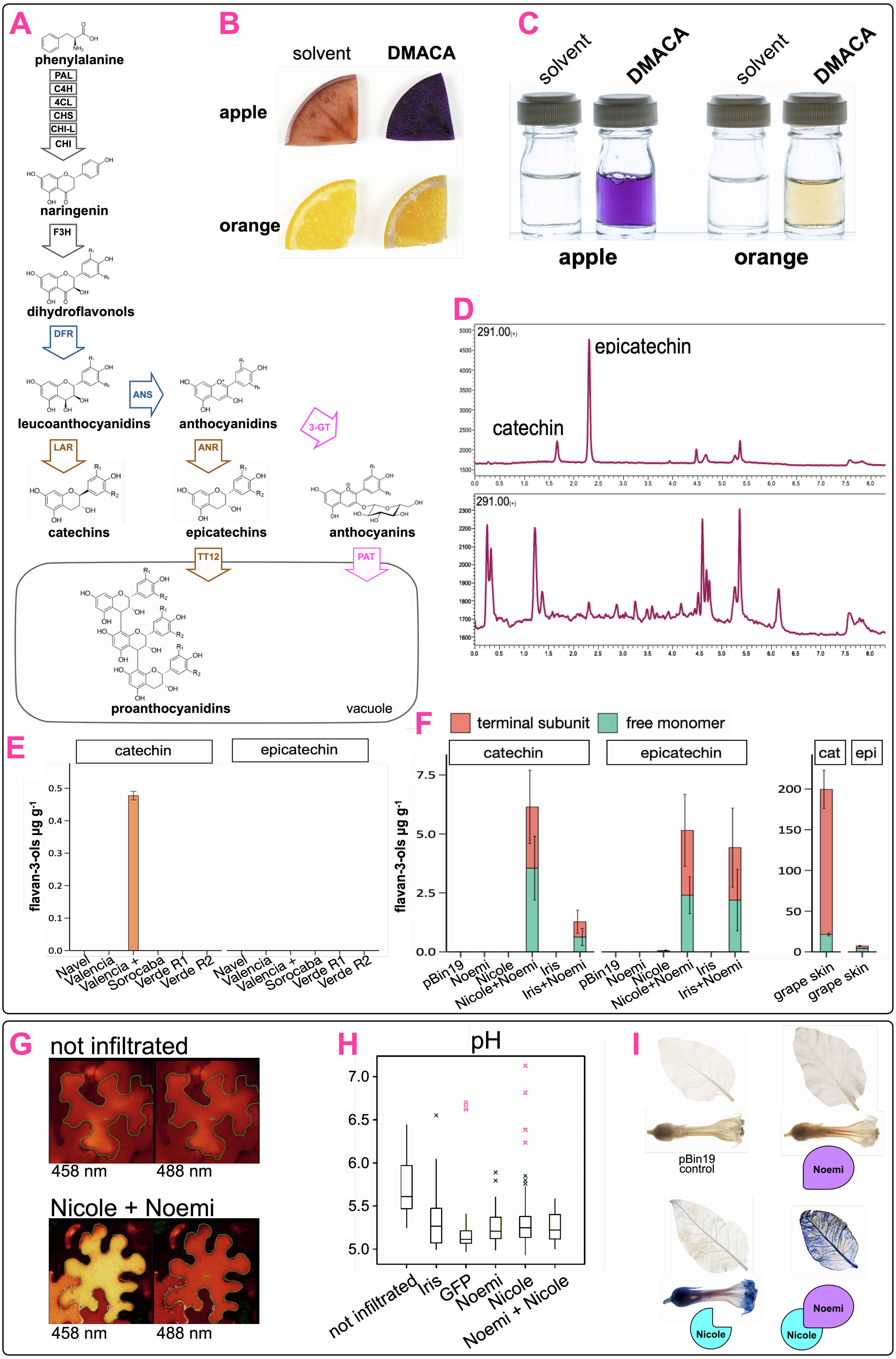
A
phenylalanine
PAL
C4H
4CL
CHS
CHI-L
CHI
naringenin
F3H
dihydroflavonols
DFR
leucoanthocyanidins
ANS
anthocyanidins
3-GT
LAR
ANR
catechins
epicatechins
anthocyanins
TT12
PAT
proanthocyanidins
vacuole
B
solvent
DMACA
apple
orange
C
solvent
DMACA
solvent
DMACA
apple
orange
D
291.00
epicatechin
catechin
291.00
E
catechin
epicatechin
flavan-3-ols µg g-1
Navel
Valencia
Valencia +
Sorocaba
Verde R1
Verde R2
F
terminal subunit
free monomer
catechin
epicatechin
cat
epi
flavan-3-ols µg g-1
pBin19
Noemi
Nicole
Nicole+Noemi
Iris
Iris+Noemi
grape skin
G
not infiltrated
458 nm
488 nm
Nicole + Noemi
458 nm
488 nm
H
pH
not infiltrated
Iris
GFP
Noemi
Nicole
Noemi + Nicole
I
pBin19
control
Noemi
Nicole
Noemi
Nicole

Figure S3.

Figure S3. *Nicole* and *Noemi* induce the accumulation of PAs (absent in flesh and juice of sweet orange) when expressed in tobacco without significant changes in pH, related to Figure 2, 3 and 4.

(A) Simplified diagram of the proanthocyanin (PA) and anthocyanin biosynthetic pathways. Genes specific for PAs are indicated by brown arrows; genes specific for anthocyanins are indicated in magenta; genes required for both anthocyanins and PAs but not for other classes of flavonoids are indicated in blue. Upstream genes involved in the biosynthesis of different classes of flavonoids are in grey. Abbreviations: PAL, phenylalanine ammonia lyase; C4H, cinnamate 4-hydroxylase; 4CL, 4-coumaroyl-CoA ligase; CHS, chalcone synthase; CHI-L, chalcone isomerase-like; CHI, chalcone isomerase; F3H, flavanone 3-hydroxylase; DFR, dihydroflavonol reductase; ANS, anthocyanidin synthase; LAR, leucoanthocyanidin reductase; ANR, anthocyanidin reductase; ANS, anthocyanidin synthase; TT12, multidrug and toxic compound extrusion protein (MATE) Transparent Testa 12; 3-GT, flavonoid 3-O-glucosyltransferase; PAT, putative anthocyanin MATE transporter. (B) Slices of apple and orange with or without staining with DMACA reagent. (C) Apple and orange juice after centrifugation incubated with solvent (1:1 6 M HCl:50 % methanol) or DMACA reagent. Fruit and juice samples were incubated for 24 hours, but the positive purple-blue colour was visible in apple almost instantly and remained undetectable in orange. Fruits and juices were obtained commercially. (D) Mass spectrometry (MS) spectra of the monitored value *m/z* 291 (+), a typical fragment ion detected in the mass spectrum of catechin and epicatechins (PA monomers). Upper panel, standards; lower panel, 'Navel' orange juice. (E) HPLC quantification of catechin and epicatechin monomers in juice of different sweet orange varieties. Catechin was detectable only in the 'Valencia +' sample, where the standard was added prior to extraction. Error bars represent the standard error of the mean (n=3 except for 'Navel', n=2). (F) HPLC quantification of catechin and epicatechin monomers in leaves of *N. tabacum* expressing different regulatory genes from citrus and in grape skin extracts as a positive control. Error bars represent the standard error of the mean (n≥3); Asterisks indicate comparative statistical significance: ∗ $p < 0.05$. (G) Representative examples of epidermal cells of *N. benthamiana* leaves with or without agroinfiltration with *Nicole* and *Noemi*; samples were treated with the cell-permeable dye 6-carboxyfluorescein diacetate (6-CFDA) and imaged after excitation at 458 nm and 488 nm. (H) pH calculated as ratio of emission intensities at different excitation wavelengths in leaves infiltrated with different constructs. Any reduction in pH was associated with the infiltration procedure and not with the type of construct introduced. (I) Transgenic tobacco plants constitutively expressing *Nicole* or *Noemi* under the control of the *CaMV 35S* promoter. Leaves and flowers were destained overnight in a solution of ethanol-glacial acetic acid (3:1, v/v) and stained with 0.3% DMACA in methanol-6M HCl (1:1, v/v) to show the presence of PAs. Plants expressing both *Nicole* and *Noemi* were obtained by crossing; they displayed stunted growth and never reached the flowering stage.

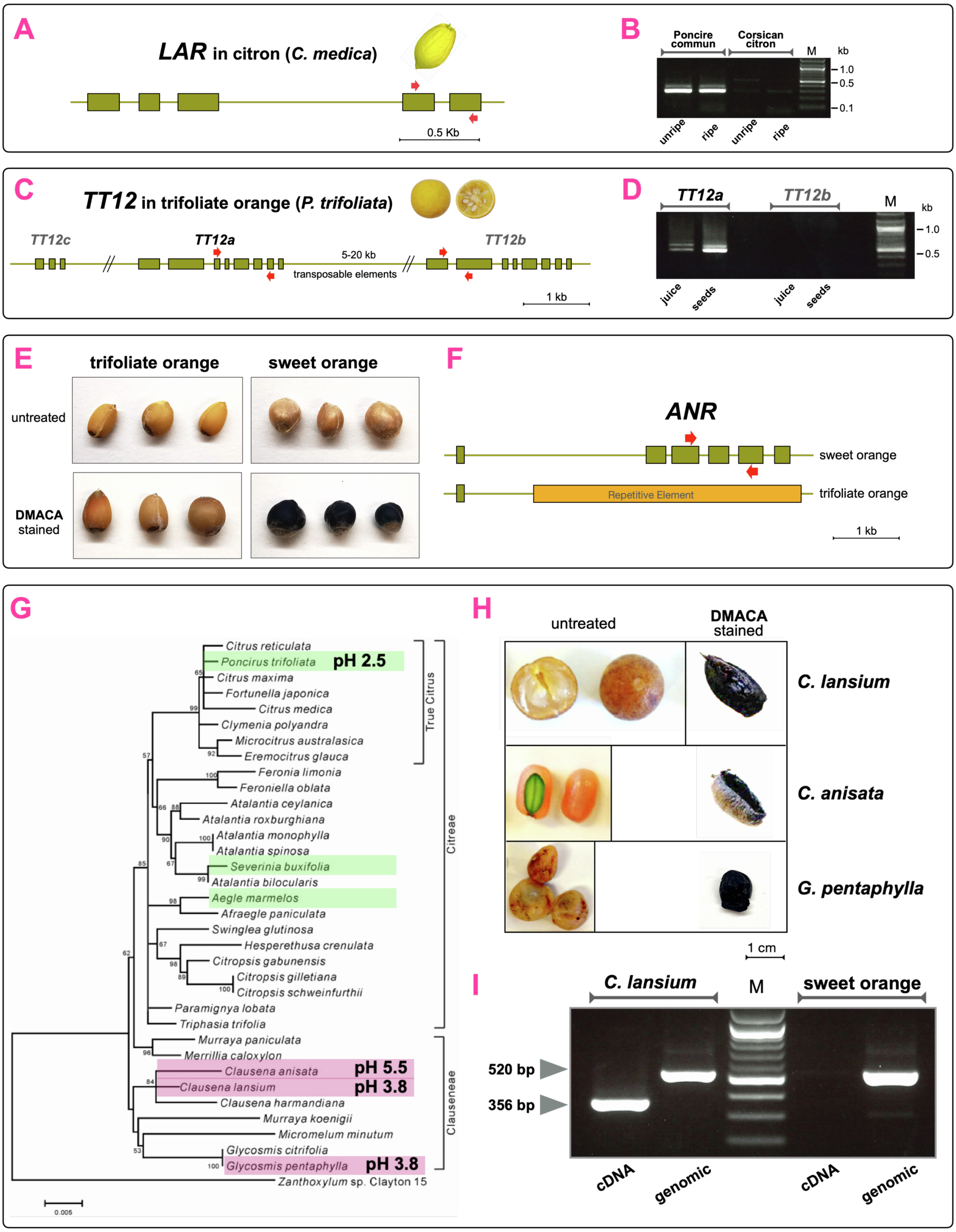

A
LAR in citron (C. medica)
0.5 Kb
B
Poncire commun
Corsican citron
M
kb
1.0
0.5
0.1
unripe
ripe
unripe
ripe
C
TT12 in trifoliate orange (P. trifoliata)
TT12c
TT12a
TT12b
5-20 kb
transposable elements
1 kb
D
TT12a
TT12b
M
kb
1.0
0.5
juice
seeds
juice
seeds
E
trifoliate orange
sweet orange
untreated
DMACA stained
F
ANR
sweet orange
Repetitive Element
trifoliate orange
1 kb
G
Citrus reticulata
Poncirus trifoliata
pH 2.5
Citrus maxima
Fortunella japonica
Citrus medica
Clymenia polyandra
Microcitrus australasica
Eremocitrus glauca
Feronia limonia
Feroniella oblata
Atalantia ceylanica
Atalantia roxburghiana
Atalantia monophylla
Atalantia spinosa
Severinia buxifolia
Atalantia bilocularis
Aegle marmelos
Afraegle paniculata
Swinglea glutinosa
Hesperethusa crenulata
Citropsis gabunensis
Citropsis gilletiana
Citropsis schweinfurthii
Paramignya lobata
Triphasia trifolia
Murraya paniculata
Merrillia caloxylon
Clausena anisata
pH 5.5
Clausena lansium
pH 3.8
Clausena harmandiana
Murraya koenigii
Micromelum minutum
Glycosmis citrifolia
Glycosmis pentaphylla
pH 3.8
Zanthoxylum sp. Clayton 15
True Citrus
Citreae
Clauseneae
0.005
H
untreated
DMACA stained
C. lansium
C. anisata
G. pentaphylla
1 cm
I
C. lansium
M
sweet orange
520 bp
356 bp
cDNA
genomic
cDNA
genomic

Figure S4.

Figure S4. *ANR* is the only gene essential for PA biosynthesis which is not expressed in fruit of any citrus species; fruits of distant citrus relatives express *ANR*, produce PAs, and have a higher pH than citrus fruit, related to Figure 4.

(A) Schematic linear map of *LAR* in citrus. (B) RT-PCR analysis of cDNA isolated from fruit of two citron varieties at two different stages of ripening. 'Poncire commun' is a wild type acidic citron from which the 'aclidless' (*noemi* mutant) 'Corsican' was derived [12]. (C) Schematic linear map of the *TT12* locus in citrus. *TT12a* is predicted to encode a functional MATE transporter in all citrus species. *TT12b* contains large deletions in citron and mandarin but not in pummelo. Several short deletions within the predicted coding sequence of *TT12b* are present in trifoliate orange. *TT12b* is not expressed in citrus. A third sequence, *TT12c*, 430 kb upstream of *TT12a*, is a pseudogene. (D) RT-PCR analysis of cDNA isolated from juice and seeds of trifoliate orange. The upstream functional gene, *TT12a,* is expressed in both tissues. The lower band corresponds to the predicted correct transcript, the upper band corresponds to a transcript with retention of the sixth intron. (E) Seeds of trifoliate orange do not produce PAs and are DMACA-negative, unlike seeds of sweet orange shown as a control. (F) Schematic linear map of the *ANR* locus in sweet orange and trifoliate orange, where the insertion of a repetitive element is associated with the deletion of most of the coding sequence. Red arrows indicate the approximate localization of the primers used for RT-PCR. M; 1 kb plus DNA ladder (NEB). (G) Neighbor-joining tree of the *matK* genes from accessions belonging to the Aurantioideae subfamily reproduced from the study of Penjor et al [37]. The pH measurements of fruit reported in this study are also shown. (H) Fruits of *C. lansium*, *C. anisata* and *G. pentaphylla* before and after staining with DMACA reagent. (I) RT-PCR analysis of cDNA isolated from fruit showing the expression of *ANR* in *C. lansium* but not in sweet orange. For both species, genomic DNA was used as a control. M; 1 kb plus DNA ladder (NEB).

Data S1. Sequence of the mutated allele *nicole^S*, related to Figure 1.

The coding sequence is indicated in turquoise, the retrotransposon Tcs7x in grey with the identical long terminal repeats (LTRs) of 549 bp in dark grey, the 7bp target site duplication is underlined at both ends of the insertion, the premature stop codon within the retrotransposon is indicated in red.

>*nicole^S*

ATGAGGAACCCATCAACATCACCATCATCAACAGCAGCAGCAGCAGCAGCAGCAGCAACCAATAAGAGCACGCCATGTT
GCAGCAAGGTAGGGTTAAAGAGAGGGCCATGGACGCCAGAGGAAGACGAGCTTCTGGCCAACTACATCAATAAAGAAGG
CGAAGGCCGGTGGCGAACTCTGCCAAAACGGGCCGGATTGCTCCGCTGCGGCAAGAGTTGCCGGCTTCGTTGGATGAAC
TATCTGAGACCCTCCGTTAAACGAGGACATATCGCCCCTGATGAAGAAGATCTCATTCTTCGCCTACATCGCCTTCTCG
GTAACCGGTAATAAGAATATAATAACCCACGAATACCTTAGGGTTTCCATGGATTTTATTTACTCGGTTTGTTTTTGGT
TTGTACTTCAGAGTTAGGGCTAGGGTTTTTGTTAACTGAATTTGGTTTTGTGTTTGTGCTTTATAGATGGTCTCTGATA
GCGGGGAGGATTCCGGGGAGAACAGATAATGAGATAAAGAATTACTGGAACACTCACCTGAGTAAGAAGCTGATAAGCC
AAGGGATTGATCCAAGAACTCATAAGCCATTGAATCAAGAACTTGATCCTTCTTCTGCTGATCAAGTTACTAATAGCAA
CAGCAAAGCTTCAACTTCGAAAGCAATACTAAACTCGAGCAGCTCAAACCCTAATCTCACTCCAATGACCGTTTCATCT
GGTCATTTAGATCAACGTCATACCTCTGCTGGCTGTGGTAGAATGATCTCGTCGATCATGATGATCAATAAGGAAAATG
GGTATTCACCGAACGCTTTAGTTGATGATCATGACAGTGAGTATCATCAAAATGGGATGATGGAGAACCCGTATACGAG
TTTATCGAATTGTGATCATCATCATGACGATGATGGGGGGTTGGGTTTGAGAAGCAATAACGTGAATAACGTTTTTAAC
GAAGGGCTTAGCTATGAAGTTGATGTAGATATCAATTACTGCAACGACGATGTTTTCTCTTCGTTTCTCAATTCGTTGA
TCAATGAAGATGCTTTTGCTAGCCAGCATAATCAACAAGTACTGCAACAACTGTCAAAGTTTTTGGCTGCAACTTCTAG
AAGCTACATCTAGAAGCGTTGATGCAGCAAATGTTGAAGGGACCGCCAAGGAAGAATGAAGAAGATGTTGCAGAAGTTG
ATCTTGACAAATGATGGTTGAAACGCCAAAATGGAAGAAAGAAAAATGTTGGAACACGGCTTTGAAGAAGCCACGAGAA
GAATAAAGAAAATATTAAAATAATAGCCAATTTTGGCTATATAAAGAAAGCTCCCCATTTTTGGTTTTTTGCATCTAAT
CCTCGGCTTCTTTTCTTCATTCAGAGAGTATTTCTTTGGGGTGTATTTGGGGCTTAGGTGAGAGAAAATTATTTCTGAG
AGTGTGGTTGTAATAATTTTCCACATAGTGAATATTTTTTCTCTGGTTGTCTTTTTGACAACGGCCGTGGTTTTTCTCC
GGATTTGGAGTTTTCCACGTAAATCTTGTGTTGTGTGATTGGTGTATTCTCCATTAAATTTTTTCTGTTAATTTGTTGC
TTGACAAATTGCTTAGAGTGATCTTGGGAGGAAGCTCAATTTCCTAACAGTGGTATCAGAGCCATTGATTTAAGTTTTG
GTGTTGGGGCACTGTTCACGTATACGGTACTGTTCACGTATACGGTACTATTCACGTGAAGCAGTGGGAGCCAATCCAA
ACAGTCTGTGGTGAAGAGTAAAAGCTTATCTGCAAAGCAAATATGTCAGGATTGAAATTTTCAAGTCCGGTGAAATTTG
AAATAGAAAAATTCGATGGGAGAATTAACTTTGGCTTGTGGCAAGTTCAAGTCAAAGATGTGTTAATTCAATCTGGGTT
ACACAAGGCATTGAAGGGGAAGCCATCCCCTGCTTCCAGTAGTGGCTCTGGAAAAACTAGTATAAGTGATGAAGATTGG
GAAGAATTAGATGATAGAGCTGCAAGTGCCATACGACTGTGTCTAGCAAAGAATGTTCTTGCAAATGTAGGAAAAATTC
CTACAGCGAAAGAACTTTGGGAGAAGCTAGAAAAGTTGTATCAGACAAAGAGCATCTCAAATCGATTGTACCTGAAGGA
GCGATTTCACACACTGCGAATGGCTGAAGGTACAAAAATTTCCGATCACCTCAGTGTTCTCAATGGTATTGTGTCAGAA
CTAGAAGCCATTGGAGTTAAAATTGAAGATGAGGACAAGGCGCTTAGGTTACTATGGTCACTTCCAACTTCCTACAAAC
ACTTGTTACCTACTTTGATGTATGGGAAGGAGACAGTAGATCTTGAAGAAGTTACTAGTACTTTACTCTCAGAAGAAAG
GAGACTGGGTGGTGAAAGTACTAAAACTATAGATGTCTCGGCTTTGGCAGTTGTAGGGAATTGGCAGAAAGATAAATCT
AAGAAGAAAGGAGTCTGCTGGGGGTGTGGACAATCGGGGCACTTAAAAAGAGATTGTCATAGTAGAAATGGAGCAGGAT
CGGCAAGTGGCTCCAGATCAGATACTGATAGTATTGCTAGTGGTAAGTCTCTCATCATCGTGGGAGACGATGATCCCTT
GTAAAATGGATGATGATGACATCCTCATGGTATACCGCTAGTACCATGAAAGGGGATATGTTACTACTAGCGGGTCCAC
AAGATTTACACACAAGGCATGGTTGGCATTGATGCAGGGTGTGTGGTGGAATTTATGTCGATGGCTGACAAACTTCCAG
GAAGGCCAACATGGAAGTTGCACCATAAATTTCAGCAGGATATTTCGACATGTGCCGACGTAAAATTCTTAGAATTGGT
AATTAATTCTAAGTGGTATACTCTTTTATGGTGGGGTATGATAATTCTCTATGGTGAGGAAAATAATAAACTTGGTGTG
AAGATTGATTGGTTCTCAATCAAATCTCCAAGTGGGAGAATGTCAAAGTTTTTGGCTGCAACTTCTAGAAGCTACATCT
AGAAGCGTTGATGCAGCAAATGTTGAAGGGACCGCCAAGGAAGAATGAAGAAGATGTTGCAGAAGTTGATCTTGACAAA
TGATGGTTGAAACGCCAAAATGGAAGAAAGAAAAATGTTGGAACACGGCTTTGAAGAAGCCACGAGAAGAATAAAGAAA
ATATTAAAATAATAGCCAATTTTGGCTATATAAAGAAAGCTCCCCATTTTTGGTTTTTTGCATCTAATCCTCGGCTTCT
TTTCTTCATTCAGAGAGTATTTCTTTGGGGTGTATTTGGGGCTTAGGTGAGAGAAAATTATTTCTGAGAGTGTGGTTGT
AATAATTTTCCACATAGTGAATATTTTTTCTCTGGTTGTCTTTTTGACAACGGCCGTGGTTTTTCTCCGGATTTGGAGT
TTTCCACGTAAATCTTGTGTTGTGTGATTGGTGTATTCTCCATTAAATTTTTTCTGTTAATTTGTTGCTTGACAAATTG
CTTAGAGTGATCTTGGGAGGAAGCTCAATTTCCTAACAACAACAACAACAGCAGCACCTATCAAATGAGACGATTGCAT
TGCCGAATACAATTACTGGCTCATCATCGGATCCTTTGGTTTCGACTGCAGCGGCATCAACTTTTGGCCTTGAAGCAAA
CTGGGAATCTCCAATCATGGCTTCTTCTTTGAACCAAGATGAGTCCAGGAGGGTTGATGAACACGTTGAGTAG

Data S2. Genomic sequence of *ANR* in sweet orange (*C. sinensis*) and *C. lansium*, related to Figure 3.

Multiple sequence alignment of the two *ANR* alleles in sweet orange (ANR^1 and ANR^2) with the *ANR* gene in *C. lansium*. The promoter regions immediately upstream of the start codon used for the transactivation assay are boxed in green. The coding sequences are boxed in red.

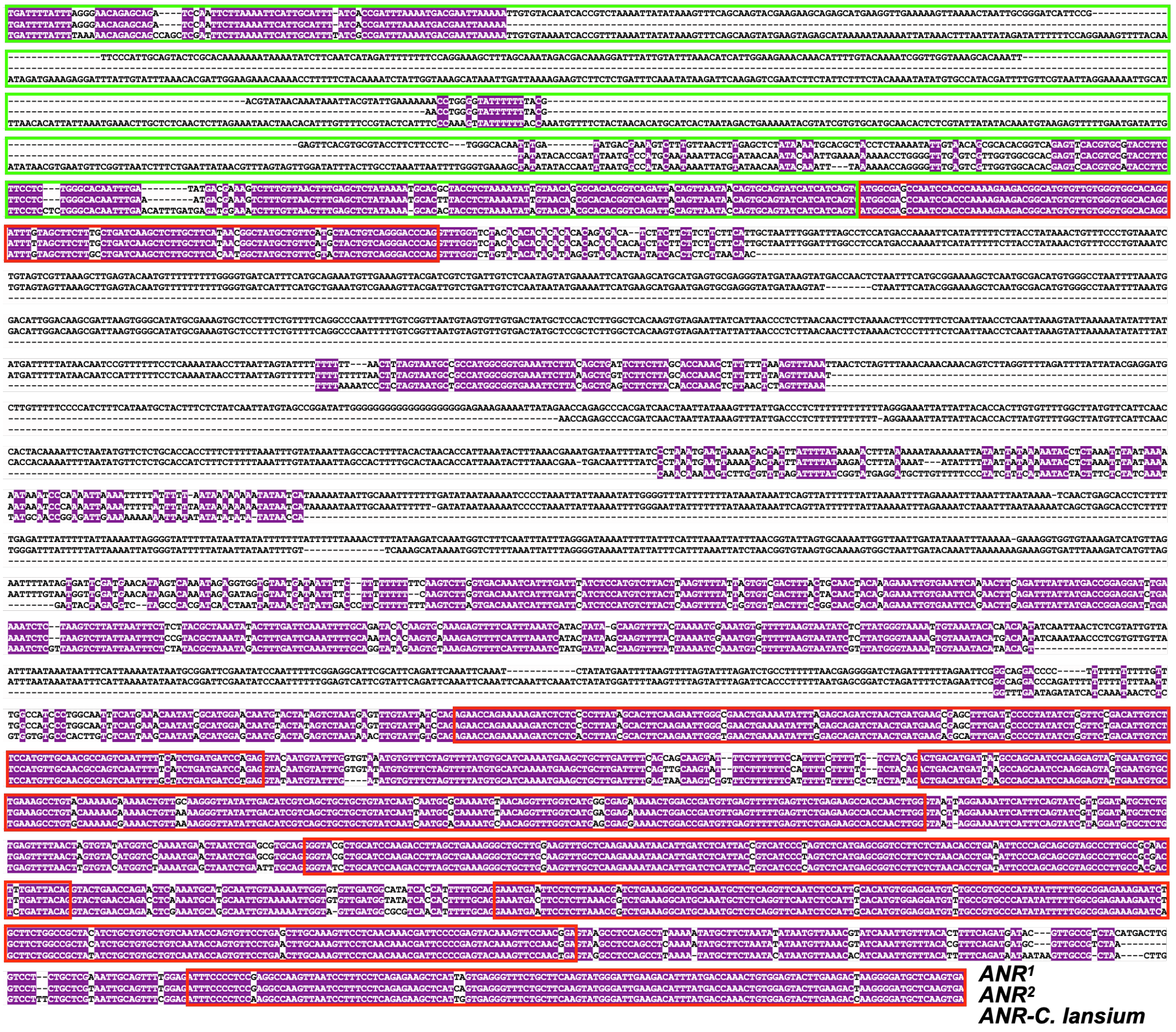

Table S1. List of *C. sinensis* and of *N. tabacum* gene accessions used for expression and phylogenetic analyses, related to Figures 1, 2 and 3.

When genes are not annotated in the *C. sinensis* v1.1 Phytozome genome, the accessions are reported according to Wu *et al.* [23].

For each *N. tabacum* gene, GenBank accession numbers for the two species that gave origin to allotetraploid tobacco, *N. sylvestris* (S-subgenome) and *N. tomentosiformis* (T-subgenome) are provided.

SWEET ORANGE (*C. sinensis*)			
**ID Phytozome v 1.1**	**Names in this study**	**Nomenclature in the phylogenetic analysis**	**Other designations**
orange1.1g040726m	*Nicole*	CsR2R3MYB46	*PH4, CrMYB73*
orange1.1g037798m	*Noemi*		*CitAN1, TT8*
orange1.1g038533m	*WDR*		*TTG1*
orange1.1g040726m	*Nicole*	CsR2R3MYB46	*PH4, CrMYB73*
orange1.1g040841m	*Iris*	CsR2R3MYB47	
orange1.1g040653m	*tt2*		*TT2* (contains mutations)
orange1.1g025602m	*Marys*	CsR2R3MYB30	
orange1.1g036591m	*Aris*	CsR2R3MYB37	
not annotated	*Ruby*		JAHMIT010000006 REGION: 22315210..22316890
orange1.1g002768m	*PH5*		*AHA10, CsPH8*
orange1.1g045750m	*PH1*		
orange1.1g018369m	*ANS*		*LDOX*
orange1.1g040253m	*DFR*		
orange1.1g038074m	*ANR*[1]		JAHMIS010000002 REGION: 29019786..29023199
orange1.1g035985m	*ANR*[2]		JAHMIT010000002 REGION: 28790149..28791461
orange1.1g046061m	*TT12a*		
orange1.1g029607m orange1.1g044433m	*TT12b*		
orange1.1g037649m	*TT12c*		
not annotated	*CHI-L*		JAHMIT010000004 REGION: 2136556..2137588
orange1.1g000304m		CsR2R3MYB1	
orange1.1g006420m		CsR2R3MYB2	
orange1.1g013300m		CsR2R3MYB3	
orange1.1g014135m		CsR2R3MYB4	
orange1.1g014829m		CsR2R3MYB5	
orange1.1g016021m		CsR2R3MYB6	
orange1.1g016708m		CsR2R3MYB7	

orange1.1g017727m		CsR2R3MYB8	
orange1.1g018154m		CsR2R3MYB9	
orange1.1g018559m		CsR2R3MYB10	
orange1.1g018746m		CsR2R3MYB11	
orange1.1g019307m		CsR2R3MYB12	
orange1.1g019787m		CsR2R3MYB13	
orange1.1g019911m		CsR2R3MYB14	
orange1.1g020197m		CsR2R3MYB15	
orange1.1g020603m		CsR2R3MYB16	
orange1.1g020613m		CsR2R3MYB17	
orange1.1g020997m		CsR2R3MYB18	
orange1.1g021188m		CsR2R3MYB19	
orange1.1g021220m		CsR2R3MYB20	
orange1.1g021486m		CsR2R3MYB21	
orange1.1g021756m		CsR2R3MYB22	
orange1.1g021816m		CsR2R3MYB23	
orange1.1g022439m		CsR2R3MYB24	
orange1.1g023056m		CsR2R3MYB25	
orange1.1g023196m		CsR2R3MYB26	
orange1.1g024441m		CsR2R3MYB27	
orange1.1g024492m		CsR2R3MYB28	
orange1.1g024849m		CsR2R3MYB29	
orange1.1g026855m		CsR2R3MYB31	
orange1.1g028843m		CsR2R3MYB32	
orange1.1g028922m		CsR2R3MYB33	
orange1.1g035629m		CsR2R3MYB34	
orange1.1g036215m		CsR2R3MYB35	
orange1.1g036344m		CsR2R3MYB36	
orange1.1g037024m		CsR2R3MYB38	
orange1.1g037956m		CsR2R3MYB39	
orange1.1g037998m		CsR2R3MYB40	
orange1.1g039016m		CsR2R3MYB41	
orange1.1g039070m		CsR2R3MYB42	
orange1.1g039198m		CsR2R3MYB43	
orange1.1g039708m		CsR2R3MYB44	
orange1.1g040502m		CsR2R3MYB45	
orange1.1g041081m		CsR2R3MYB48	
orange1.1g041991m		CsR2R3MYB49	
orange1.1g042846m		CsR2R3MYB50	
orange1.1g043269m		CsR2R3MYB51	
orange1.1g043557m		CsR2R3MYB52	
orange1.1g043612m		CsR2R3MYB53	
orange1.1g044864m		CsR2R3MYB54	
orange1.1g045384m		CsR2R3MYB55	
orange1.1g045387m		CsR2R3MYB56	
orange1.1g045400m		CsR2R3MYB57	
orange1.1g045411m		CsR2R3MYB58	
orange1.1g045434m		CsR2R3MYB59	
orange1.1g045583m		CsR2R3MYB60	
orange1.1g046075m		CsR2R3MYB61	
orange1.1g046093m		CsR2R3MYB62	
orange1.1g046419m		CsR2R3MYB63	
orange1.1g047101m		CsR2R3MYB64	
orange1.1g047269m		CsR2R3MYB65	
orange1.1g047839m		CsR2R3MYB66	
orange1.1g047854m		CsR2R3MYB67	
orange1.1g048224m		CsR2R3MYB68	

TOBACCO (*N. tabacum*)		
**Gene Name**	**S-subgenome**	**T-subgenome**
PH5	XM_009784485	XM_033657755
PH1	XM_009773363	XM_009602397
ANS	XM_009777817	XM_009605813
DFR	XM_009761654	NM_001302522
ANR	XM_009786976	XM_009632192
TT12	XM_009778895	XM_009590295
3-GT	XM_009773776	XM_009599711
PAT	XM_009779423	XM_009615762

Supplementary Table 2. Sequences of primers used in this study, related to STAR Methods.

Name	Sequence	Description
PH4-FA	CACCGCCAACAAGAAGATAACAAGACGA	Isolation of *Nicole* in sweet
PH4-RZ	ACTCGAAGGCTACTCAACGTGTTCATCA	orange
EB-832	GCCTACATACGATCATATTAAAACCCT	Identification of SNP
EB-834	GTGTCATAATTTGATTGCCTCGAGTGA	downstream of *Nicole*
EB-039	CATGGACGCCAGAGGAAGACGA	Detection of *Nicole* alleles in
PH4-RZ	ACTCGAAGGCTACTCAACGTGTTCATCA	Figure S2A
EA-209	CCAAGCAGCATGAAGATCAA	*actin*-qPCR sweet orange
EA-210	ATCTGCTGGAAGGTGCTGAG	
EA-215	CGGTAACCGATGGTCTCTGA	*Nicole*-qPCR sweet orange
EA-216	TCCCTTGGCTTATCAGCTTCT	
EA-217	CTTCCGGAGTTGGGTTACCA	*Noemi*-qPCR sweet orange
EA-218	TCCTCCGGGACCTTTTCTGT	
E79	ATGGTCTCTCATCGCAGGGA	*Iris*-qPCR sweet orange
E80	GTGGGTATTCGGGTCGGTTC	
EA-233	ACCCACCCACCAAGCTAATG	*WDR*-qPCR sweet orange
EA-234	GAGCGCAAAACTCACTGCTC	
EA-245	GTGGTTCACTTTCTGGTTTGAAG	*WRKY*-qPCR sweet orange
EA-246	TTGGTTTGCACTGTAGGTTGTC	
EA-223	CTTTGGAAAAGCAGCACA	*PH5*-qPCR sweet orange
EA-224	ATCCCTGTATGATCGATGCT	
EA-221	CGAAACACCTGGTAGTAAGCC	*PH1*-qPCR sweet orange
EA-222	ACATTTGTGCCCATGAAGCAG	
EA-229	ACTCCAAGCGACTATACAGAGG	*ANS*-qPCR sweet orange
EA-230	TCCCAAGCCAAGTGACAACA	
EA-235	AGATGACTGGATGGATGTATTTTGTGT	*DFR*-qPCR sweet orange
EA-236	TGAGACTGGGTGGCATTGAC	
EB-913	ATGCTCTTGTCTCTGCTCCA	*CHI-L*-qPCR sweet orange
EB-914	ACTTGGATTGGAAGAATTCAACGA	
EA-251	CTCTCCCCTTATAGCACTTCAAG	*ANR*-qPCR sweet orange
EA-252b	TGGCWTAATCATGTCAGTCTCTG	
EA-249	GCTGTCATGCTTTGTTTGGAG	*TT12a*-qPCR sweet orange and
EA-250	GACAGCCCCAACATAAATTGC	*P. trifoliata*
EB-940	GCTAAAACAGGCCTCAATAGATGTGGA	*Aris*-conventional RT-PCR
EB-941	ACTATTTGCCTTGCTCTCTTCGCT	sweet orange
EB-942	CCCAAAGCTGCAGGTCTTAAGAGA	*Marys*-conventional RT-PCR
EB-943	ATCGTCATCAGCAACACCGCCA	sweet orange
EB-944	GGCTGGGCTTCTTAGATGTGGA	*Iris*-conventional RT-PCR sweet
EB-945	TCTTGTGGGTATTCGGGTCGGT	orange

EB-858	ATGAGCGAGGAAAACTGGACCGA	*ANR*-conventional RT-PCR *C. lansium*
EB-889b	CAGATATAGCGGCCAGAAGCA	
EB-859	ATCCTGAGACTGACATGATCAAGCCA	*ANR*-conventional RT-PCR *C. lansium*
EB-860	GGAACTTTGTACTCGGGGAATCGT	
EB-705	CATGGACGAGAAAAACTGGACCGA	*ANR*-conventional RT-PCR sweet orange
EB-889	CAGATGTAGCGGCCAGAAGCA	
EB-879	GACAGTTCATTTTAGGCCACCCT	*LAR*-conventional RT-PCR citron
EB-880	GAGATAATCACTGAAGCAATCATCCA	
EB-752	ATCAAGGACTGGTGCTTATATCTGGA	*TT12a*-conventional RT-PCR sweet orange and *P. trifoliata*
EB-753	ACTCCGATAATCATCCCCCACCA	
EB-747	GCTAGCGTGGGGATTCAGGGT	*TT12b*-conventional RT-PCR sweet orange and *P. trifoliata*
EB-748	CTATGCTTTCAGATTGCCCCATTGCT	
EB-882	CCCCTCACCACAGAGTCTGC	*L25 ribosomal protein gene*-qPCR tobacco
EB-883	AAGGGTGTTGTTGTCCTCAATCTT	
NIF1	AGGACATATCGCCCCTGATG	*Nicole*-qPCR tobacco
NIR1	ATCTGTTCTCCCCGGAATCC	
NOF1	CACAACAGAAAAGGTCCCGG	*Noemi*-qPCR tobacco
NOR1	TGTGAGTGAAAACGCAGGTG	
IRF1	TTCTAAGGTTGGGTTGCAGC	*Iris*-qPCR tobacco
IRR1	GCCTGCAACTCTTTCCACAT	
EB-922	ATACGAATTGTGCTTGGTTTCATGCT	*PH5*-qPCR tobacco
EB-923	AGCTTCCAACTGTCGGGTCCT	
E37	GTGTCATGCTCGTTGTGGTT	*PH1*-qPCR tobacco
E38	GAAGCATCTGAGGGGTGAGT	
E15	GAGAAGGAAGTCGGAGGCAT	*ANS*-qPCR tobacco
E16	CTTTGCCGTTACCCACTGTC	
E21	CCAAAGGCAGAGGGGAGATT	*DFR*-qPCR tobacco
E22	CCCCATATCCGTCAGCTTCT	
E9	TTTGCTCTGCCATCAACACC	*ANR*-qPCR tobacco
E10	GATGATCAGCTTGGCCTTGG	
E17	TGTGGCTGTTGGTAGTGGAT	*TT12*-qPCR tobacco
E18	ACCCCACCATATTCCTGCTT	
E27	GGACGTGAAGCCATTGAGC	*3-GT*-qPCR tobacco
E28	AGAACCAGAACCAGCAGTCC	
E29	GTTGGGGTACTACTGGTGCT	*PAT*-qPCR tobacco
E30	AATCTCATTAAACGCCGCCC	
EB-117	GGGGACAAGTTTGTACAAAAAAGCAGGCTTAATGAGGAACCCATCAACATCACCA	Gateway cloning of wild type *Nicole* CDS
EB-118	GGGGACCACTTTGTACAAGAAAGCTGGGTACTACTCAACGTGTTCATCAACCCT	
PH4-FA	CACCGCCAACAAGAAGATAACAAGACGA	Isolation of mutated *nicole*^{S} CDS
EB-628	CTTCAACATTTGCTGCATCAACGCT	
EB-117	GGGGACAAGTTTGTACAAAAAAGCAGGCTTAATGAGGAACCCATCAACATCACCA	Gateway cloning of mutated *nicole*^{S} CDS
EB-629	GGGGACCACTTTGTACAAGAAAGCTGGGTCTTCAACATTTGCTGCATCAACGCT	

EB-115	GGGGACAAGTTTGTACAAAAAAGCAGGCTATGGATGCTCCGCCGCCGAGTA	Gateway cloning of *Noemi* CDS
EB-116	GGGGACCACTTTGTACAAGAAAGCTGGGTGAGTGAGTTAATTGACATACTGGGGT	
EB-382	GGGCTCCCTGTTGTTCTAAGGT	Isolation of *Iris* CDS
EB-384	CTGATCATTTTAGAAGAGATTCAGCA	
EB-394	GGGGACAAGTTTGTACAAAAAAGCAGGCTATGGGAAGGGCTCCCTGTTGTTCT	Gateway cloning of *Iris* CDS
EB-397	GGGGACCACTTTGTACAAGAAAGCTGGGTTCATTTTAGAAGAGATTCAGCAAAGCA	
EB-693	GCTAATGGCTTCAAYGATGGA	Isolation of *Aris* CDS
EB-694	AAGAAGCATTACCTAAAGCCCT	
EB-698	GGGACAAGTTTGTACAAAAAAGCAGGCTGCTAATGGCTTCAAYGATGGA	Gateway cloning of *Aris* CDS
EB-699	GGGGACCACTTTGTACAAGAAAGCTGGGTAGAAGCATTACCTAAAGCCCT	
EB-378	GGAGAGGAAAAGTTGTTAAGAGTCA	Isolation of *Marys* CDS
EB-380	GATTCAAGAAAAGGTCACCAAAATCCCA	
EB-543	GGGGACAAGTTTGTACAAAAAAGCAGGCTATGGCGGGTAAGCGCAAGA	Gateway cloning of *Marys* CDS
EB-544	GGGACCACTTTGTACAAGAAAGCTGGGTCAAGAAAAGGTCACCAAAATCCCA	
EB-677	CAAAAATGGAAAACTCAAGCCAAGA	Isolation of *WDR* CDS
EB-678	GATCAAACTTTCAAAAGTTGCATTTTGT	
EB-683	GGGGACAAGTTTGTACAAAAAAGCAGGCTCAAAAATGGAAAACTCAAGCCA	Gateway cloning of *WDR* CDS
EB-684	GGGGACCACTTTGTACAAGAAAGCTGGGTGATCAAACTTTCAAAAGTTGCATT	
EB-422	ATGAGAACCCCATCATCATCATCAACA	Isolation of *PhPH4* CDS
EB-423	CTAACTGGGATTATATTGATCAGATGAAGGT	
EB-424	GGGGACAAGTTTGTACAAAAAAGCAGGCTATGAGAACCCCATCATCATCATCA	Gateway cloning of *PhPH4* CDS
EB-425	GGGGACCACTTTGTACAAGAAAGCTGGGTCTAACTGGGATTATATTGATCAGATGA	
EB-426	ATGCAGCTGCAAACCATGTTACGGA	Isolation of *PhAN1* CDS
EB-427	CTTAAACTCTAGGGATTAACTGGTGT	
EB-428	GGGGACAAGTTTGTACAAAAAAGCAGGCTATGCAGCTGCAAACCATGTTACGGA	Gateway cloning of *PhAN1* CDS
EB-429	GGGGACCACTTTGTACAAGAAAGCTGGGTCTTAAACTCTAGGGATTAACTGGTGT	
EB-510	AGATAGCTCGAGGAGGGAAAGACCGTTAGGT	Isolation of sweet orange *pPH5* for transactivation assay
EB-511	AGATAGACAAGCGGCCGCGACTGGAGTTTGAAATCTCAACCT	
EA-204	CGAGCTGCAGGGATTGACCTTTGAGTGC	Isolation of sweet orange *pPH1* for transactivation assay
EA-199	TCTTCCATGGTGAACAAATTGTTTTATTGAATGA	
EB-508	AGATAGCTCGAGAAGTAAAACGACATTTCATCGA	Isolation of sweet orange pANS for transactivation assay
EB-509	AGATAGACAAGCGGCCGCCGTAGCAAWTTTTTGTATATATGA	
EB-651	AGATAGCTCGAGCAAGTTTTGCAAGTAAGGCCTTATTCT	Isolation of sweet orange *pDFR* for transactivation assay
EB-648	AGATAGACAAGCGGCCGCGGACTACTGATACTAAATGTTTGATCA	
EB-662	GAATAGCTCGAGGTTATCTATATATGAAGGTAGACT	Isolation of sweet orange *pTT12a* for transactivation assay
EB-663	AGATAGACAAGCGGCCGCGACTAATGTTAGTCACTAAGGTGT	
EB-725	GATTCGGTACCTGATTTTATTTAGGGAACAGAGCA	Isolation of sweet orange *pANR* for transactivation assay
EB-727	AGCATGCCATGGACTGATGATGATACTGCACTGT	
EB-876	GATTCGGTACCTGATTTTATTTTAAAAACAGAGCAGCCA	Isolation of *C. lansium pANR* for transactivation assay
EB-727	AGCATGCCATGGACTGATGATGATACTGCACTGT	
